# The Impact of Company-Level ART Provision to a Mining Workforce in South Africa: A Cost–Benefit Analysis

**DOI:** 10.1371/journal.pmed.1001869

**Published:** 2015-09-01

**Authors:** Gesine Meyer-Rath, Jan Pienaar, Brian Brink, Andrew van Zyl, Debbie Muirhead, Alison Grant, Gavin Churchyard, Charlotte Watts, Peter Vickerman

**Affiliations:** 1 Department of Health Services Research and Policy, London School of Hygiene & Tropical Medicine, London, United Kingdom; 2 Center for Global Health and Development, Boston University, Boston, Massachusetts, United States of America; 3 Health Economics and Epidemiology Research Office, Department of Internal Medicine, Faculty of Health Sciences, University of the Witwatersrand, Johannesburg, South Africa; 4 Highveld Hospital, Anglo American Coal, Emalahleni, South Africa; 5 Anglo American, Johannesburg, South Africa; 6 The Aurum Institute, Johannesburg, South Africa; 7 Department of Clinical Research, London School of Hygiene & Tropical Medicine, London, United Kingdom; 8 Department of Global Health and Development, London School of Hygiene & Tropical Medicine, London, United Kingdom; 9 School of Social and Community Medicine, University of Bristol, Bristol, United Kingdom; Harvard School of Public Health, UNITED STATES

## Abstract

**Background:**

HIV impacts heavily on the operating costs of companies in sub-Saharan Africa, with many companies now providing antiretroviral therapy (ART) programmes in the workplace. A full cost–benefit analysis of workplace ART provision has not been conducted using primary data. We developed a dynamic health-state transition model to estimate the economic impact of HIV and the cost–benefit of ART provision in a mining company in South Africa between 2003 and 2022.

**Methods and Findings:**

A dynamic health-state transition model, called the Workplace Impact Model (WIM), was parameterised with workplace data on workforce size, composition, turnover, HIV incidence, and CD4 cell count development. Bottom-up cost analyses from the employer perspective supplied data on inpatient and outpatient resource utilisation and the costs of absenteeism and replacement of sick workers. The model was fitted to workforce HIV prevalence and separation data while incorporating parameter uncertainty; univariate sensitivity analyses were used to assess the robustness of the model findings. As ART coverage increases from 10% to 97% of eligible employees, increases in survival and retention of HIV-positive employees and associated reductions in absenteeism and benefit payments lead to cost savings compared to a scenario of no treatment provision, with the annual cost of HIV to the company decreasing by 5% (90% credibility interval [CrI] 2%–8%) and the mean cost per HIV-positive employee decreasing by 14% (90% CrI 7%–19%) by 2022. This translates into an average saving of US$950,215 (90% CrI US$220,879–US$1.6 million) per year; 80% of these cost savings are due to reductions in benefit payments and inpatient care costs. Although findings are sensitive to assumptions regarding incidence and absenteeism, ART is cost-saving under considerable parameter uncertainty and in all tested scenarios, including when prevalence is reduced to 1%—except when no benefits were paid out to employees leaving the workforce and when absenteeism rates were half of what data suggested. Scaling up ART further through a universal test and treat strategy doubles savings; incorporating ART for family members reduces savings but is still marginally cost-saving compared to no treatment. Our analysis was limited to the direct cost of HIV to companies and did not examine the impact of HIV prevention policies on the miners or their families, and a few model inputs were based on limited data, though in sensitivity analysis our results were found to be robust to changes to these inputs along plausible ranges.

**Conclusions:**

Workplace ART provision can be cost-saving for companies in high HIV prevalence settings due to reductions in healthcare costs, absenteeism, and staff turnover. Company-sponsored HIV counselling and voluntary testing with ensuing treatment of all HIV-positive employees and family members should be implemented universally at workplaces in countries with high HIV prevalence.

## Abstract 2

Gesine Meyer-Rath and colleagues assess whether workplace ART provision can be cost-saving for mining companies in high HIV prevalence settings.

## Editors' Summary

**Background:**

Every year, more than 2 million people become newly infected with HIV, the virus that causes AIDS, usually by having unprotected sex with an infected partner. People in the early stages of HIV infection rarely have any symptoms, but, over time, HIV destroys CD4 lymphocytes and other immune system cells, and, eventually, HIV-positive individuals become susceptible to numerous other infections. Because many of these infections are extremely serious, early in the AIDS epidemic, most HIV-infected individuals died within ten years of infection. Then, in 1996, effective antiretroviral therapy (ART)—cocktails of drugs that stop HIV replicating—became available. For people living in affluent countries, HIV/AIDS became a chronic condition, but because ART was expensive, HIV/AIDS remained fatal in low- and middle-income countries. In 2003, the international community began to work towards achieving universal access to ART. By 2013, nearly 13 million HIV-positive people—more than a third of the global HIV-infected population—had access to ART.

**Why Was This Study Done?:**

HIV disease hits individuals in the prime of their working lives, thereby increasing absenteeism, the turnover of labor, and the operating costs of companies working in countries where HIV infection is common (high HIV prevalence). To reduce the economic impact of HIV/AIDS, some companies provide their workforces in such countries with comprehensive HIV services that include counseling and testing, and ART. For example, mining companies in South Africa (where nearly 20% of the working-age population is HIV-positive) provide HIV services to their workforces. However, although there is strong evidence that HIV disease increases the cost of doing business, a full cost–benefit analysis (the quantification of both the costs and benefits of a business strategy or medical intervention) of ART provision in the workplace based on real-world data has not been undertaken. Here, the researchers use a mathematical model to estimate the economic impact of HIV and the costs and benefits of company-level ART provision by a South African mining company between 2003 and 2022.

**What Did the Researchers Do and Find?:**

The researchers developed a mathematical model to evaluate the past and future impact and costs to the employer of an ART program provided since 2002 by a coal mining company operating at a number of South African colleries. They fed data on the workforce’s characteristics, the annual number of new HIV infections in the workforce, the CD4 cell counts of HIV-positive employees, healthcare resource utilization, and the costs of absenteeism and labor turnover into the model. The model estimated that, as ART coverage increased from 10% to 97% of eligible employees, increases in the survival and retention of HIV-positive employees and reductions in absenteeism and benefit payments would lead to overall cost savings compared to a scenario of no ART provision. Specifically, the annual cost of HIV to the company would decrease by 5% and the average cost per HIV-positive employee would decrease by 14% by 2022. These changes in costs (which mainly accrue from reductions in benefit payments for death and ill-health retirement and in employee healthcare costs) translate into average savings of nearly US$1 million per year. Finally, scaling up ART coverage through a universal test and treat strategy would double savings, whereas providing ART for family members as well as employees would reduce savings but remain marginally cost-saving compared to no ART.

**What Do These Findings Mean?:**

These findings suggest that workplace ART provision can be cost-saving for companies operating in settings with a high HIV prevalence because of reductions in healthcare costs, absenteeism, and staff turnover. That is, the costs to the employer of providing ART can be less than the costs saved by reducing healthcare use, absenteeism, and worker turnover. The accuracy of these findings depends on the quality of the data used to run the model. However, additional analyses indicate that ART provision is likely to be cost-saving unless people receive no benefits on leaving the workforce or the absenteeism rate is considerably lower than the available data suggest. Thus, the researchers propose that company-sponsored counseling and voluntary HIV testing with treatment of all HIV-positive employees and family members should be implemented universally at workplaces in countries with a high HIV prevalence. Such a strategy should be cost-saving for employers and might also take some pressure off resource-limited public sector ART programs.

**Additional Information:**

This list of resources contains links that can be accessed when viewing the PDF on a device or via the online version of the article at http://dx.doi.org/10.1371/journal.pmed.1001869.

- Information is available from the US National Institute of Allergy and Infectious Diseases on HIV infection and AIDS
- NAM/aidsmap provides basic information about HIV/AIDS, summaries of recent research findings on HIV care and treatment, and personal stories about living with HIV/AIDS
- Information is available from Avert, an international AIDS charity, on many aspects of HIV/AIDS, including information on ART, universal access to ART, and HIV/AIDS in South Africa; Avert also provides personal stories about living with HIV/AIDS
- The World Health Organization provides information on all aspects of HIV/AIDS (in several languages), including its guidelines on the use of ART for treating and preventing HIV infection
- The UNAIDS Fast-Track Strategy to End the AIDS Epidemic by 2030 provides up-to-date information about the AIDS epidemic and efforts to halt it; UNAIDS also provides detailed information about HIV/AIDS in South Africa
- Wikipedia has a page about cost–benefit analysis (note that Wikipedia is a free online encyclopedia that anyone can edit; available in several languages)
- The HIV Modelling Consortium has a database of models used in analyzing the impact of HIV and ART, including models such as the one used in this analysis
- The International AIDS Economics Network has a library of research on the economics of HIV around the world

## Introduction

HIV disease hits adults in the prime of their working lives. Companies therefore take a heavy toll in countries with high HIV prevalence [1,2]. To counter this, some companies provide their workforce with a number of HIV services, ranging from prevention activities to HIV testing and antiretroviral therapy (ART).

While several companies in sub-Saharan Africa started ART programmes from 2002 onwards [3–5], quantifying these programmes’ costs and benefits has proven difficult [3]. Even in sophisticated in-house medical programmes, longitudinal data collection is fraught with difficulty, and the relationship between costs and benefits, such as regained productivity, can be hard to establish [3]. This makes it hard for companies to plan and budget for additional HIV-specific health programmes, and impossible to ascertain the programme’s impact on the company’s operations and profits.

HIV disease increases rates of absenteeism, labour force turnover, and, ultimately, the costs of company operations in sub-Saharan Africa. A number of studies have quantified the impact of HIV on labour forces in the region, with the cost of HIV ranging from 0.7% of wages [6] or 1% of labour cost [7] to 1%–9% of profits [8]. Only one study, amongst Kenyan tea pluckers, has estimated the impact of HIV on the productivity of a single worker, finding an 18% decrease in earnings in the year before termination amongst HIV-positive workers [9], in a setting where earnings are directly related to productivity.

South Africa is the sub-Saharan African country with the largest number of people living with HIV [10,11], with 18.8% of the working-age population (15–49 y old) being HIV infected [12]. In the last large-scale survey of 22 companies in South Africa, between 1999 and 2005, the workforce HIV prevalence in a non-representative sample averaged 11% [13], though estimates varied over time and between industries [3,13]. Similarly, the costs of HIV vary, with the estimated increase due to HIV in the cost of doing business (termed AIDS “tax” [1]) ranging from 0.4% to 5.9% of the annual wage bill of six South African companies in 2001 [1,2], or a 0.6%–10.8% increase in labour costs amongst companies from six countries in sub-Saharan Africa [3]. The cost per employee also varies considerably by skill level [2]. None of these studies, however, included the impact of workplace ART provision.

HIV care, including ART, has been provided by mining companies in South Africa since 2002, predating ART provision in the public sector [4,5]. While there are numerous estimates of the cost [14–27] and cost-effectiveness [28–45] of public sector ART provision in South Africa, the cost and impact of private sector ART provision at the workplace level have not yet been established. And while some aspects of this impact have been estimated in other countries, such as Kenya [46–50], Botswana [50], and Uganda [51], none of these estimates included productivity as well as healthcare costs, and none was a full cost–benefit analysis based on real-world programme data. In order to provide evidence for company management and policy-makers alike, we evaluated the impact and cost of both HIV and ART in a mining company in South Africa, and analysed the incremental cost–benefit balance of the company’s ART programme compared to no ART provision.

## Methods

### Workplace under Study

We report on the ART programme of a coal mining company operating at a number of collieries in Mpumalanga province since 2002. The programme is run from the mines’ own clinics and hospitals and provides care for employees, contractors, and employees’ dependants. Annual anonymous HIV counselling and testing (HCT) campaigns in the mines provide easy access to testing. HIV-positive employees are enrolled in an HIV wellness programme that provides CD4 cell count testing every 3 mo and interventions, such as isoniazid and cotrimoxazole prophylaxis, for the prevention and treatment of opportunistic infections. Employees were initiated on ART once their CD4 cell count was at or below 250 cells/mm^3^ during the period 2003–2007, or at or below 350 cells/mm^3^ during 2008–2010, or if presenting with WHO stage 3/4 disease, and their CD4 cell count and viral load (VL) were monitored twice annually thereafter. By the end of 2010, out of 9,252 employees, 1,149 had tested HIV positive in confirmatory tests and had been enrolled in the company’s wellness programme. Since 2002, 629 employees have been initiated on ART, with 555 employees retained on ART by the end of 2010.

### Model Description

**Fig 1:**
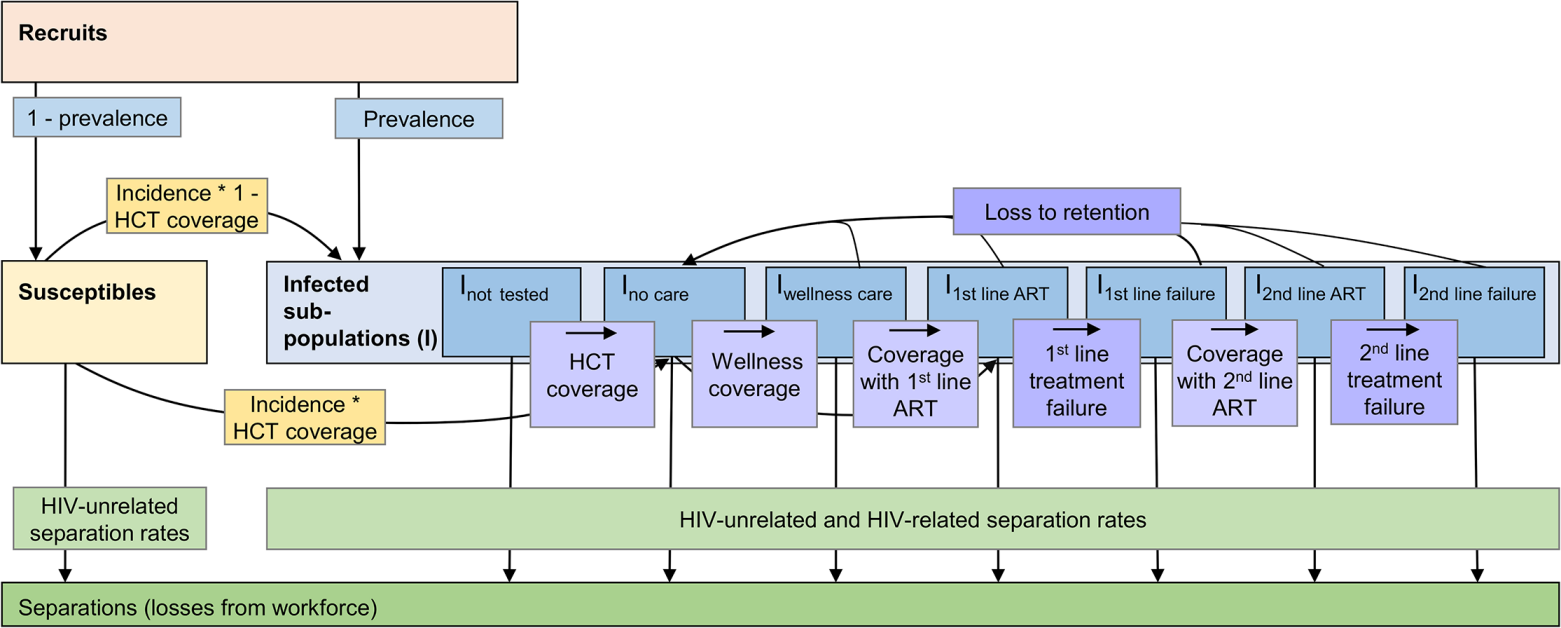
Population model of changes within the workforce. Recruits join the susceptible or infected (*I*) workforce depending on their HIV status at first employment. Employees move from the susceptible to the infected population according to prevalence and incidence. In the infected population, employees change between sub-populations representing different types of care (not tested, tested but not yet in care, wellness care, successful first- or second-line ART, and first-line or second-line treatment failure) according to coverage rates and, in case of treatment failure, to failure rates. Employees can drop out of care, i.e., be lost to retention, at any time and go back to the no care sub-population according to loss-to-retention rates; they can also leave the workforce for reasons related or unrelated to HIV (separations). Within each of the sub-populations, additional unidirectional changes due to ageing and promotion rates apply (not shown here); within each of the infected sub-populations, additional bi-directional changes due to transitions between CD4-cell-count-defined health states apply.

A dynamic Markov health-state transition model, the Workplace Impact Model (WIM), was developed to evaluate both the past and future impact and costs of introducing ART into the workforce from the perspective of the employer. The model is run twice, under a scenario of no ART provision (no ART scenario) and again under a scenario representing the scale-up of ART in the workforce (ART scenario). Both scenarios also include the cost and impact of other components of HIV healthcare such as HIV testing, wellness care, and other outpatient and inpatient care for HIV. The model projects the HIV-positive and-negative workforce over 20 y from 2003, taking into account planned changes to the workforce size as well as ageing and promotions. This time period is necessary to capture the full impact of the gradual scale-up of ART. The model calculates, in 3-mo time steps, employees’ HIV prevalence, their HIV test uptake and coverage with and loss from wellness and ART care, the number of employees leaving the workforce as a result of mortality and morbidity due to HIV (separations), the number of recruits to the workforce (some of which are HIV infected) that are required to offset this loss, the change in CD4 cell count (an indicator of immune system function) in HIV-positive employees, and the incremental costs of the ART programme itself, of additional outpatient and inpatient healthcare, and of absenteeism and workforce turnover (Fig 1).

In order to capture important differences in survival and/or in healthcare and absenteeism costs, the HIV-infected workforce is divided into two genders, three age groups, six job grades, and five CD4-cell-count-defined health states, although not every parameter is differentiated by all four categories. Table 1 summarises the population categories used in the model; Table 2 gives more detail on the stratification levels.

**Table 1:** Job grade, health state, and age group categories used in model.

Parameter	Category
**Patterson band** ^**1**^	
A	Job grade 1 (unskilled worker)
B lower	Job grade 2 (semi-skilled worker)
B upper	Job grade 3 (semi-skilled worker)
C lower	Job grade 4 (skilled worker)
C upper	Job grade 5 (skilled worker)
D and E	Job grade 6 (management)
**CD4 cell count stratum (cells/mm** ^**3**^ **)**	
>350	Health state 1
200–350	Health state 2
100–199	Health state 3
50–99	Health state 4
<50	Health state 5
**Age range (years)**	
<30	Age group 1
30–50	Age group 2
>50	Age group 3

^1^South African system of grading jobs according to the level of skill required for a certain job.

**Table 2:** Details of parameter estimation, level of stratification, and data sources.

Model Input or Assumption	Level of Stratification	Source of Data (2003–2010)	Method of Estimation	
			2003–2010^1^	2011–2022
**1. Changes in workforce**				
Workforce needed at end of year	Job grade, year	Company data	Data taken as is to calculate number of recruits or retrenchments required	Assumed to remain same as in 2010
Number of recruits	Job grade, year		Set to produce workforce needed at end of year	Same as for 2003–2010
Prevalence of recruits/retrenchees^2^	Job grade, gender, year (for retrenchees, also by age)	Company data	N: all new employees with a positive first HIV result in the year of recruitment; D: all new employees with a positive or negative first HIV result in the year of recruitment	Assumed to remain same as in 2010
Distribution of recruits	Age group, gender, year	Company data (distribution set to be same as workforce distribution in database in 2003–2010)	N: number of employees in database by year, job grade, gender, and age group; D: total number of employees across all job grades, age groups, and genders by year	Assumed same as average 2003–2010
Annual rate of promotion	Job grade, year	Company data for 2005/2006	Assumed to remain same as in 2005/2006	Assumed to remain same as in 2005/2006
**2. Start population and coverage**				
Distribution of start population (all employees)	Age group, gender, job grade	Company data	Number of employees in database by 31 Dec 2002 by job grade, gender, and age group	N/A (start year only)
HIV status of start population (all employees)	HIV status of those employees with an HIV test	Company data	Number of HIV-positive employees tested before 31 Dec 2002 and assumptions regarding untested employees’ HIV status	N/A (start year only)
Distribution of start population into CD4 cell count categories (HIV-positive employees)	CD4 cell count category	No data	Same proportion assumed in each CD4 cell count strata	N/A (start year only)
Baseline HCT coverage^3^	Age group, gender, job grade	Company data	Number of employees tested before 31 Dec 2002 by job grade, gender, and age group	N/A (start year only)
**3. Costs**				
Average basic salary	Job grade	Company data (payroll)	Salaries in cost year (2006)	Real cost assumed constant over time
Incremental replacement cost for HIV-positive employees	Job grade	Interviews with company human resources department	Average cost per new employee by job grade in cost year (2006)	Real cost assumed constant over time
Number of years that benefits get paid	None	Company benefit policy	Company policy	Real cost assumed constant over time
Incremental inpatient/outpatient cost for HIV-positive employees in cost year (2006)	Type of care (ART/no ART), CD4 cell count category	Bottom-up cost analysis of company health services	Average cost per employee in cost year (2006); includes non-ARV drugs, non-ARV-specific laboratory tests, patient contact time, other medical supplies, site programme cost, but no central management cost	Real cost assumed constant over time
Annual per employee cost of ART in cost year (2006)	CD4 cell count category	Bottom-up cost analysis of company health services	Average cost per employee in cost year (2006); includes central management cost for ART programme, ARV drug cost, ART-specific laboratory tests (CD4, VL)	Real cost assumed constant over time
Incremental absenteeism cost for HIV-positive employees	Type of care (ART/no ART only), CD4 cell count category, job grade	Payroll data on sick leave days	Absent days/shifts lost to sickness (sick leave) by health state in cost year (2006) multiplied by job-grade-specific salary per day/shift	Real cost assumed constant over time
**4. Transitions between CD4 cell count categories**				
Transition probabilities	Type of care, CD4 cell count category	No care: public sector data based on [52]; all else: company data	N: all employees with a CD4 cell count in one stratum in time period *t* who have a CD4 cell count in a different stratum in time period *t* + 1; D: all employees with a CD4 cell count in one stratum in time period *t* that also had a CD4 cell count in time period *t* + 1	Assumed constant over time
Transition probabilities	Type of care, CD4 cell count category	No care: public sector data based on [52]; all else: company data	N: all employees with a CD4 cell count in one stratum in time period *t* who have a CD4 cell count in a different stratum in time period *t* + 1; D: all employees with a CD4 cell count in one stratum in time period *t* that also had a CD4 cell count in time period *t* + 1	Assumed constant over time
**5. HIV incidence; coverage with testing, care, and ART; and treatment failure and retention**				
Incidence	Job grade, CD4 cell count category^4^, year	Change in HIV incidence over time fitted to company data on HIV incidence [53]; job grade weights: company data; CD4 cell count category weights: assumed	HIV seroconversion was assumed to occur at the midpoint between the first positive and the last previous negative HIV test; N: all employees with a calculated seroconversion date in one year; D: all employees with a negative HIV result and no seroconversion date in the previous year. This analysis excludes employees whose HIV test result was given as “unknown”	Assumed same as average of 2008–2010
Coverage with HIV testing, wellness care, and ART	Type of care, year, and, for ART, also CD4 cell count category	Company data	Model fitted to reported proportions of HIV-positive employees in each type of care	Assumed same as average of 2008–2010, except transition to first-line ART from wellness care, which is used to achieve ~92% ART coverage of eligible population
Rate of treatment failure	Year (same for first- and second-line ART)	Company data	N: employees with a failure start date during time period *t*; D: all employees on ART at the beginning of time period *t*	Assumed same as average of 2008–2010
Loss-to-follow-up rate	Type of care, year	Company data	N: all employees with a care stop date (wellness care and ART only) during time period *t*; D: all employees in wellness care and ART, respectively, at the beginning of time period *t*	Assumed same as average of 2008–2010
**6. Separation rates**				
HIV-related	Type of separation, CD4 cell count category	Company data	Ill-health, death, and other non-transfer separations were allocated to a CD4 cell count category using the last available CD4 cell count before exit from the workforce from the database; N: all HIV-positive employees with an employment stop date by separation category and CD4 cell count category; D: all employee-years in the same CD4 cell count category	Assumed constant over time
HIV-unrelated	Type of separation, job grade	Company data	N: all HIV-negative employees with an employment stop date by separation category and job grade; D: all employee-years in the same job grade	Assumed constant over time

“Company data” refers to the mine company’s employee database of 9,211 employees and a separate database documenting the 1,149 employees who tested HIV positive and were enrolled in the company’s HIV care programme. The databases cover the period January 2003 to December 2010.

^1^Details of analysis are given if a parameter was analysed from the company’s employee database. D, denominator; N, numerator.

^2^If the workforce is set to be reduced during one year, the resulting number of recruits will be negative, signifying the number of people who will be retrenched, rather than recruited, during that year.

^3^Coverage with all other care is set to zero at baseline.

^4^Incidence is stratified by CD4 cell count category to allow the distribution of newly incident members of the infected population into CD4 cell count categories. The values of the weights are 0.1, 0.2, 0.3, 0.5, and 1 for the categories >350, 200–350, 100–199, 50–99, and <50 cells/mm^3^, respectively.

ARV, antiretroviral; N/A, not applicable.

Due to the difficulty in capturing the programme’s benefit to dependants, this analysis is limited to employees. The model incorporates HIV incidence in the workforce but does not model HIV transmission from the workforce or the effect of ART on HIV transmission. Separations, i.e., losses to the workforce other than through retirement or retrenchment, most often due to ill-health or death, are differentiated into three categories (death, ill-health/disability, and other) in the model and are further differentiated by HIV status, job grade for HIV-negative employees, and CD4 cell count stratum for HIV-positive employees. More details on the methods used in estimating each parameter are given in Tables 2–4 and in S1 Text, which also gives the model equations.

**Table 3:** Values and sources of main model inputs and assumptions.

Parameter	Value by Job Grade							Source
	1	2	3	4	5	6	Total	
**Workforce needed at end of year**								Business plans from human resource managers
2003	133	857	2,251	954	673	379	5,247	
2004	128	858	2,250	1,122	695	400	5,453	
2005	137	894	2,282	1,276	743	450	5,782	
2006	152	982	2,326	1,534	798	507	6,300	
2007	247	1,069	2,348	1,749	875	591	6,879	
2008	324	1,243	2,590	2,105	986	722	7,969	
2009	451	1,386	2,776	2,356	1,086	820	8,875	
2010 and onwards	705	1,433	2,772	2,405	1,119	818	9,252	
**Salaries and benefits in 2010 US dollars**								
Average annual basic salary	10,047	12,043	16,057	20,740	25,925	54,242	—	Human resource data
Employee benefits (ill-health and death benefit: three times annual salary)	30,141	36,128	48,171	62,220	77,775	162,726	—	Interviews with pension and provident fund administrators, document review, and claims data
Recruitment and training cost per new recruit	55,096	55,096	55,096	55,096	55,096	84,133	—	Human resource data
**HIV-unrelated separations (percent of workforce leaving per year)**								
Disability/ill-health	0.66%	0.08%	0.21%	0.24%	0.09%	0.03%	—	Workforce data
Death	0.99%	0.21%	0.57%	0.35%	0.28%	0.26%	—	
Other^1^	5.63%	1.50%	1.78%	8.53%	4.79%	5.92%	—	

^1^Other separations include dismissals in absentia.

**Table 4:** Values and sources of main model inputs and assumptions (HIV-related separations only).

HIV-Related Separations (Incremental to HIV-Unrelated Separations)	CD4 Cell Count (cells/mm^3^)				
	>350	200–350	100–199	50–99	<50
Disability/ill-health	1.20%	1.80%	2.10%	2.70%	14.00%
Death	3.00%	4.70%	9.20%	24.80%	67.10%
Other^1^	6.90%	8.20%	8.60%	9.00%	12.90%

Source: workforce data.

^1^Other separations include dismissals in absentia.

### Model Parameterisation

The model was parameterised with company data on the size, composition, and turnover of the workforce at the mines obtained from the company employee database of 9,211 employees, covering the period January 2003 to December 2010 and including job grade, gender, engagement and termination dates, and the coverage and results of the serial HCT campaigns. Annual coverage with linked workplace HCT campaigns increased from 40% of all employees in 2003 to 86% in 2008, enabling a reliable estimation of HIV incidence in later years. A separate database documenting the 1,149 employees who tested HIV positive and were enrolled in the company’s HIV care programme over the same period of time provided inputs regarding coverage of wellness care and ART, retention in care, development of treatment failure, and employees’ CD4 cell counts over time. The two databases were anonymously linked for this analysis.

We parameterised the model with annual HCT and ART coverage, HIV prevalence in new employees joining the workforce, as well as the incidence of treatment failure and loss to retention in the programme as reported in these databases. Based on these data, HCT coverage was set to reach 92% by 2010 and to remain constant thereafter. The HCT data were also used to estimate the HIV incidence and prevalence amongst all employees. Incidence was estimated for those employees with two or more HIV tests, with HIV conversion assumed to be at the midpoint between the first positive and the last prior negative HIV test [53]. These data suggested that HIV incidence varied between 1.2 and 2.6 per 100 employee-years in the workforce throughout and that prevalence increased from 11% in 2005 to 16% in 2010. ART coverage of those eligible was calibrated to increase from 11% in 2003 to 68% in 2010, as suggested by the workforce data, and was modelled to reach 88% by 2013 and 100% by 2022. First-line treatment failure was set to vary between 8% and 11% per year, and loss to follow-up between 6% and 12% per year, likely including some migration to ART programmes outside the workforce. The values of important model parameters are summarised in Tables 3 and 4; the remainder of the parameters and their 95% confidence intervals are available in S1 Text.

### Transition Probabilities

A detailed electronic register including the results of all CD4 cell count measurements (every 3 mo) from all HIV-positive employees for the same period as the workforce database (January 2003–December 2010) was used to estimate the transition probabilities between CD4-cell-count-defined health states for the wellness care and ART populations (Table 5). The database contained a total of 10,972 CD4 cell count test results, with a mean patient follow-up of 961 d (maximum 2,822 d). Since almost all employees who test HIV positive in the workplace testing programme immediately enter care, we used historic data from the South African public sector to parameterise the transitions for the undiagnosed and no care populations [52]. Because of insufficient data, these transitions were also applied to the treatment failure population.

**Table 5:** Model 3-mo transition probabilities between CD4-cell-count-defined health states by type of care.

Ending CD4 Cell Count (cells/mm^3^)	Starting CD4 Cell Count (cells/mm^3^)					Source
	>350	200–350	100–199	50–99	<50	
**Untested, no care, or treatment failure**						[52]
>350	0.94	0	0	0	0	
200–350	0.05	0.92	0	0	0	
100–199	0.01	0.06	0.94	0	0	
50–99	0.001	0.01	0.04	0.91	0	
<50	0.002	0.01	0.02	0.09	1.00	
**Wellness care**						Workforce data
>350	0.86	0.16	0.01	0	0	
200–350	0.13	0.71	0.23	0.05	0.07	
100–199	0.01	0.12	0.59	0.20	0.07	
50–99	0	0	0.14	0.55	0.14	
<50	0	0	0.04	0.20	0.71	
**First- and second-line ART**						Workforce data
>350	0.93	0.21	0.02	0	0.17	
200–350	0.07	0.74	0.28	0.03	0	
100–199	0	0.05	0.69	0.41	0.33	
50–99	0	0	0.02	0.47	0.17	
<50	0	0	0	0.09	0.33	

Each employee’s available CD4 cell count data were allocated to each type of care in 3-mo time periods from the start date for this type of care up until the time period including the stop date for this type of care. If CD4 cell counts were missing for one or two consecutive time periods, they were linearly interpolated from the CD4 cell counts of the two adjacent time periods. These CD4 cell counts were then allocated to five different CD4 cell count strata, which in turn defined the model health states (see Table 1).

For the calculation of transition probabilities, in order to differentiate between patients in wellness care and those accessing ART outside the company healthcare system, CD4 cell counts were considered to be wellness care CD4 cell counts only if any VL measured during the same 3-mo time period was unsuppressed (>50 copies/ml). If a suppressed VL count was found before the date of ART initiation in the workforce programme, the patient was deleted from the wellness care CD4 analysis. In order to exclude patients in treatment failure, CD4 cell counts were considered to be ART CD4 cell counts only if any VL measured during the same time period was suppressed (≤50 copies/ml), though the patient could still contribute other (i.e., earlier or later) CD4 cell counts to the ART CD4 population if they coincided with a suppressed VL.

### Cost Data

A bottom-up patient-level analysis of economic costs from the employer perspective was conducted in 2006 to quantify all costs of HIV/AIDS to the company. The analysis, which has been described in detail elsewhere [54,55], included the cost of the ART programme, including the cost of antiretroviral drugs, ART-specific laboratory tests such as CD4 cell count and VL, and management and training costs within and above the facility level, as well as any other HIV-related cost such as inpatient and outpatient resource utilisation and costs, and the costs of absenteeism and replacing a sick or deceased worker, including the benefits paid to the worker or his/her family and the costs of recruiting and training a replacement. Healthcare resource use, quantified as the number of inpatient days and outpatient visits, was abstracted from record systems at the company health centres and averaged by CD4 cell count stratum, based on the employee’s most recent CD4 cell count. Absenteeism was calculated as the median number of days of sick leave of patients in wellness care and on ART by CD4 cell count stratum, based on the company’s payroll data. Both healthcare and absenteeism costs were calculated incrementally to that of HIV-negative employees.

Due to the choice of an employer perspective, costs to the employee and the broader society were excluded, but since most employees of the mining company seek care at the workplace clinics and hospitals, resource use captured for this analysis is unusually complete. Cost inputs are summarised in Table 6. Cost data were collected in South African rands (ZAR) during 2006/2007, adjusted for inflation to 2010, and converted to US dollars (USD) using the 2010 average conversion rate of 8 ZAR/1 USD (S1 Text contains an explanation of the time period for inflation adjustment). Costs are presented undiscounted and discounted at 5% per annum, the repurchase rate of the South African Reserve Bank during most of the analysis period [56].

**Table 6:** Annual per employee cost and frequency of absenteeism by CD4 cell count category, incremental to that of HIV-negative employees.

Parameter	Items Included	Cost in 2010 USD by CD4 Cell Count				
		>350 Cells/mm^3^	200–350 Cells/mm^3^	100–199 Cells/mm^3^	50–99 Cells/mm^3^	<50 Cells/mm^3^
**Medical care**						
***Patients not on ART***						
Inpatient care	Mean cost of inpatient care per year	335	425	557	1,832	1,153
Outpatient care	Mean cost of outpatient care per year	164	152	157	129	250
***Patients on ART***						
Inpatient care	Mean cost of inpatient care per year	222	133	219	303	1,166
Outpatient care	Mean cost of outpatient care per year	122	124	120	124	147
ART (first and second line)	Drugs, laboratory tests, other medical supplies, staff time, site programme cost, and central management cost per year	1,826	1,826	1,826	1,826	1,826
**Absenteeism**						
***Patients not on ART***	Median days absent due to sickness per year	18	15	24	39	55
***Patients on ART***	Median days absent due to sickness per year	11	13	16	23	55

### Model Calibration and Sensitivity Analysis

Because sampling uncertainty surrounds many of the important model parameters, we defined probability distributions around the main inputs, with the distributions based on the primary workforce, absenteeism, and cost data used in this analysis. Some parameters were also stratified by CD4 cell count or job grade (separation rates) or were time dependent (treatment failure probability). Statistical distributions were assigned to these parameters based on standard practice in economic evaluations [57], with specific details included in S1 Text.

To calibrate the model while accounting for this sampling uncertainty, 20,000 parameter sets were randomly sampled (using Latin hypercube sampling) from the parameter distributions, and the resulting model runs were compared to see if they fit within the uncertainty range for the observed HIV prevalence of the workforce in 2010 (12.8%–19.2%) and the average annual number of separations in HIV-positive (50–150) and HIV-negative (200–500) employees during 2005–2009. The 998 model runs that fit these data were then used to assess the uncertainty around our main outcomes (total costs, cost savings, and HIV prevalence), with medians and 90% credibility intervals (CrIs) being produced for each outcome. In addition, an analysis of co-variance was undertaken to quantify the contribution of different parameters to the uncertainty in the projected undiscounted savings due to ART.

Additionally, we undertook univariate sensitivity analyses on selected parameters, examining the impact of the following: reducing all absenteeism by half; assuming the same absenteeism on ART as off ART; assuming the same ART cost and health-state transition probabilities as found in analyses of public sector ART provision in South Africa using similar methodology [58,59]; changing inpatient and outpatient costs by ±50% (note that in each instance only the extremes of the range were considered); changing the number of annual salary equivalents paid out as benefits to 0, 1, or 2 y instead of 3; changing HIV-dependent separation rates by ±20%; changing incidence by ±50%; and, in order to examine the generalisability of results to a setting with low HIV prevalence, reducing incidence to an extremely low value of 0.0001 and prevalence in the start population and amongst new recruits each to a tenth of the baseline values. For each of these sensitivity analyses, the effect of the parameter change was evaluated on all the baseline model fits so that an average effect could be estimated.

Lastly, in order to analyse the future impact of changes in treatment policies, we parameterised the model for two additional scenarios to be implemented from 2013 onwards. First, we considered a universal test and treat scenario in which HCT coverage was 100% each year, and 100% of employees who tested HIV-positive initiated ART within 6 mo, regardless of CD4 cell count or clinical status. We conservatively assumed no impact of this high-level ART coverage on HIV incidence since the intervention would cover only employees and not their sexual partners. In a second scenario (“family treatment”), we incorporated the extension of ART to those family members of employees who were eligible for ART, with an assumed average of one ART-eligible dependant per HIV-positive employee on ART.

### Ethics Approval

The study was reviewed and approved by the following ethics committees: the London School of Hygiene & Tropical Medicine Ethics Committee (application number 962), the Anglogold Health Service Research Ethics Committee (AHS REC 004/02), and the University of KwaZulu-Natal Biomedical Research Ethics Committee (BE093/08). Employees’ consent to participation in this study was waived as we used only data that were collected for routine care purposes and, as in most other routine care settings, employees did not give written consent for this care.

### Data Availability

The fully parameterised model that incorporates all data and that was used to produce all projections within this paper can be downloaded from OpenBU via http://hdl.handle.net/2144/10817.

## Results

### Patient-Level Cost and Resource Use and Absenteeism of Employees on and off ART

The results of our bottom-up cost analyses in HIV-positive employees show that regardless of ART status, average annual outpatient and inpatient employee costs both increase with decreasing CD4 cell count, and, in contrast to analyses of the cost of public sector ART provision in South Africa [26–29], inpatient costs are higher than outpatient costs per patient-year (Table 6). Once employees initiate ART, these costs of care decrease dramatically across all CD4 cell count strata. However, when considering the healthcare cost of the HIV programme only, and excluding other HIV-related costs such as absenteeism and the cost of staff turnover, the addition of ART renders the HIV programme more expensive than without ART.

HIV-positive employees not on ART have between 11 and 40 sick leave days annually over and above the average number of sick leave days in HIV-negative employees (Table 6). For specific CD4 strata, the level of absenteeism decreases by 16%–42% after ART initiation, except in employees with a CD4 cell count of <50 cells/mm^3^. As with healthcare costs, the most absenteeism is seen in the lowest CD4 cell count stratum, whether on or off ART.

### Coverage with Care, Survival in Employment, and HIV Prevalence

**Fig 2:**
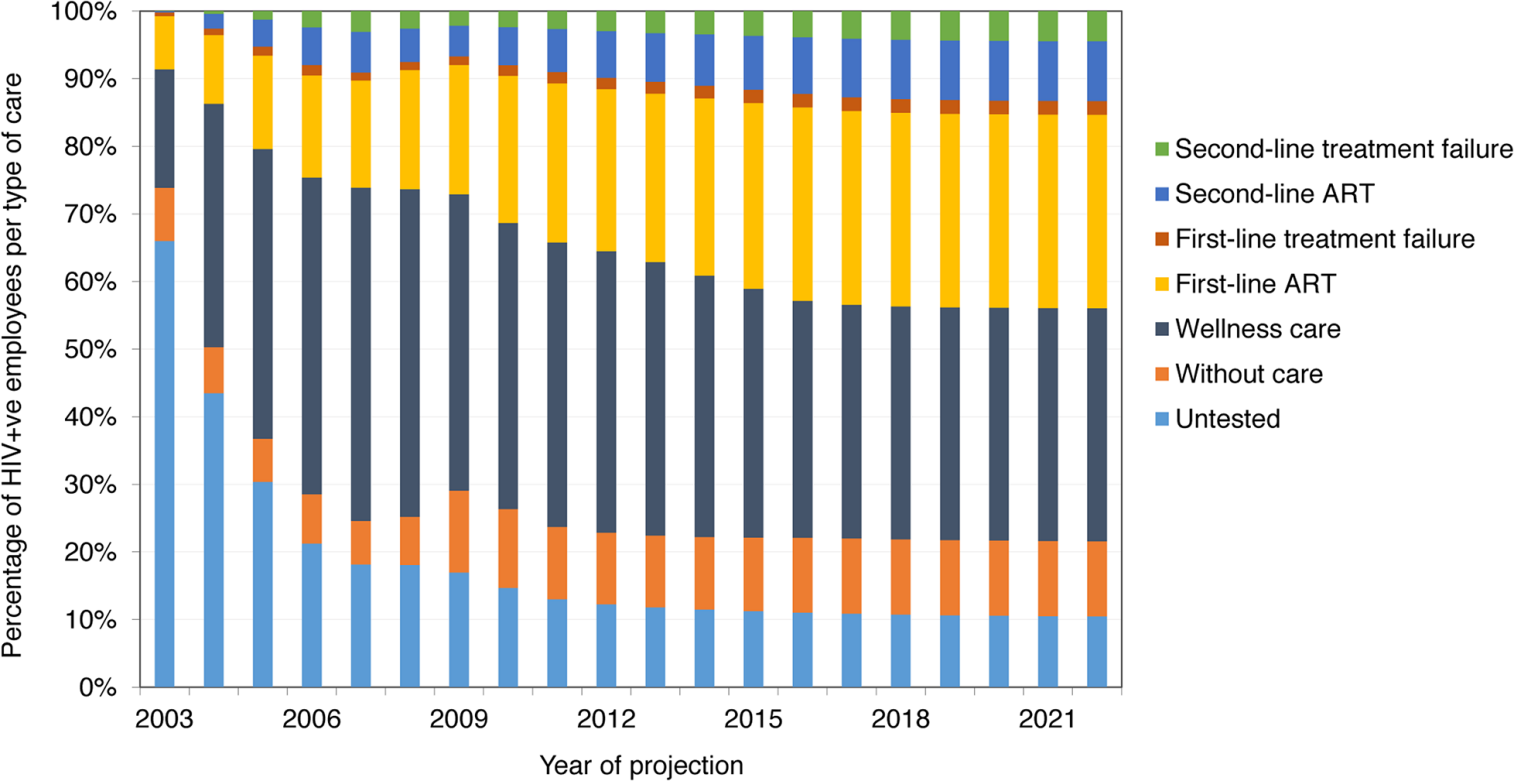
Distribution of HIV-positive employees into types of HIV care, 2003–2022 (ART scenario).

Fig 2 shows the distribution of employees into types of care over the model projection period. While the proportion of untested HIV-positive employees falls with increasing HCT coverage, the proportion in wellness care first increases and then drops slightly as the proportion of employees on ART increases. From 2010, the proportion of employees in each type of care remains relatively stable, with newly tested HIV-positive employees moving quickly through wellness care and, if eligible, onto ART, and the proportion of employees on second-line ART slowly increasing. From 2012, only 35%–44% of HIV-positive employees are on ART, because many are not eligible for ART; however, 75%–97% of employees with CD4 cell count < 350 cells/mm^3^ are on ART.

Across all available model fits, projections suggest that an HIV-infected employee with a current CD4 cell count > 350 cells/mm^3^ will have a 39% (90% CrI 35%–43%), 57% (50%–62%), or 78% (73%–82%) probability of surviving the following 10 y if they are in no care, in wellness care, or on ART, respectively. (Note that this survival does not take into account deaths in employees once they have left the workforce.) However, survival in the workforce at 10 y is much lower, as a result of death as well as disability and other separations: 16% (90% CrI 13%–19%), 23% (20%–27%), and 35% (31%–39%) for employees in no care, in wellness care, and on ART, respectively.

**Fig 3:**
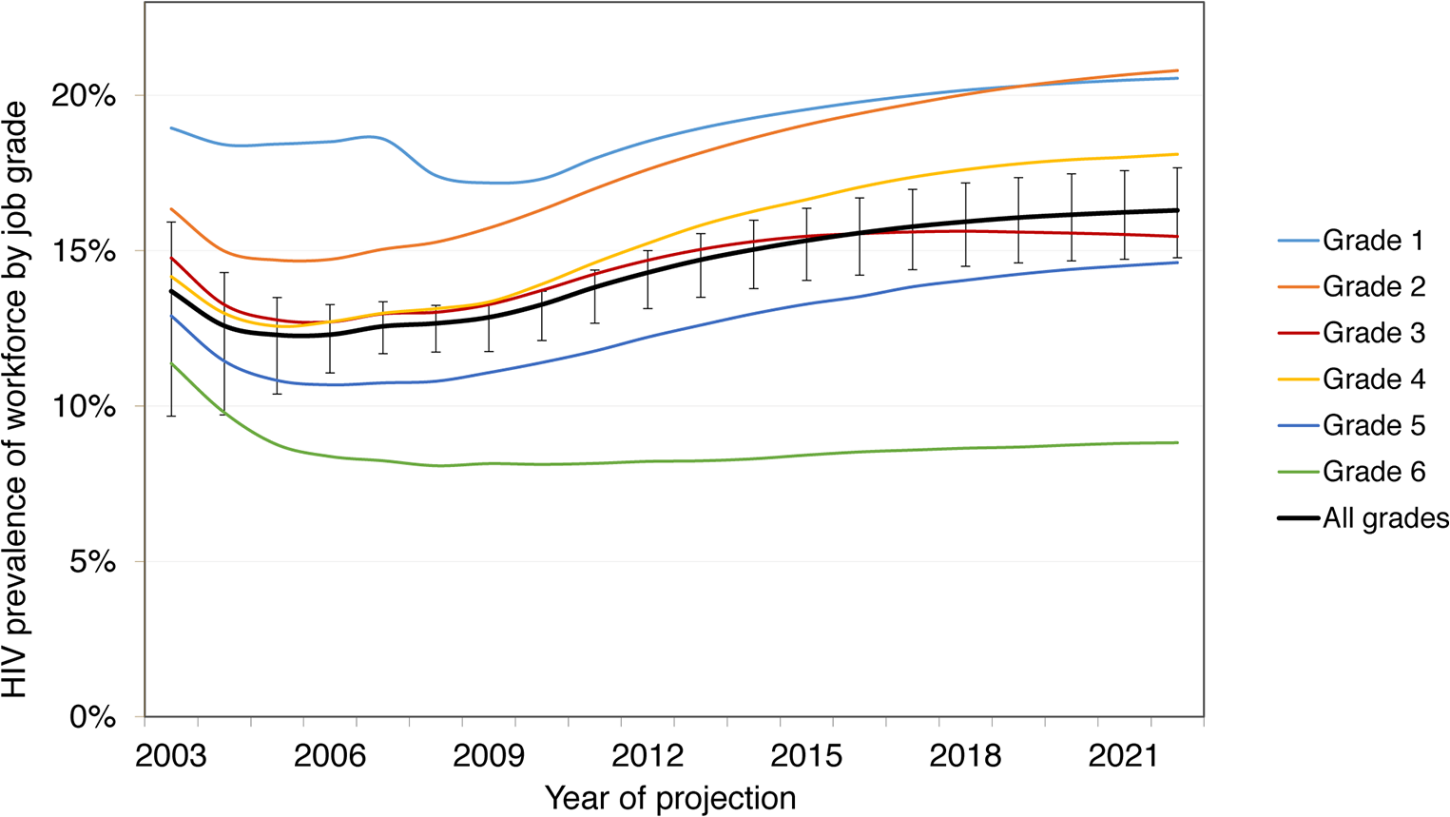
Prevalence by job grade, 2003–2022, with workplace ART provision. Job grade 1: unskilled worker; grades 2 and 3: semi-skilled worker; grades 4 and 5: skilled worker; grade 6: management.

Without ART, these survival rates lead to a total of 22,274 (90% CrI 20,887–24,086) HIV-positive employee-years (or life-years in employment) at the mines between 2003 and 2022, with HIV prevalence increasing from 13.3% (90% CrI 12.8%–14.4%) in 2010 to 14.3% (13.0%–15.9%) in 2022. With ART coverage increasing from 10% of eligible HIV-positive employees in 2003 to 97% in 2020, the number of deaths amongst employees due to HIV over 20 y decreases by 16% (90% CrI 11%–21%) from 1,583 (90% CrI 1,406–1,791) without ART to 1,336 (1,183–1,497) with ART. Survival in employment increases by 8% (90% CrI 6%–12%) to 24,134 (90% CrI 22,848–25,841) HIV-positive life-years. This increase is not larger because on average only 34% of HIV-infected employees are on ART at any given time (since only a fraction of HIV-infected employees are eligible for ART), only a portion of these would have left the workforce or died in absence of ART over this period, and some leave the workforce before realising the full benefit of treatment. The increase in survival leads to an increase in HIV prevalence from 14.3% (90% CrI 13.0%–15.9%) in 2022 without ART to 16.3% (14.9%–17.8%) with ART. HIV prevalence is always higher in the lower job grades: 21% (90% CrI 19%–22%) in job grade 1 and 21% (18%–24%) in job grade 2 in 2022 with ART (Fig 3).

### Changes in Workforce Turnover, Absenteeism, and Separations

**Fig 4:**
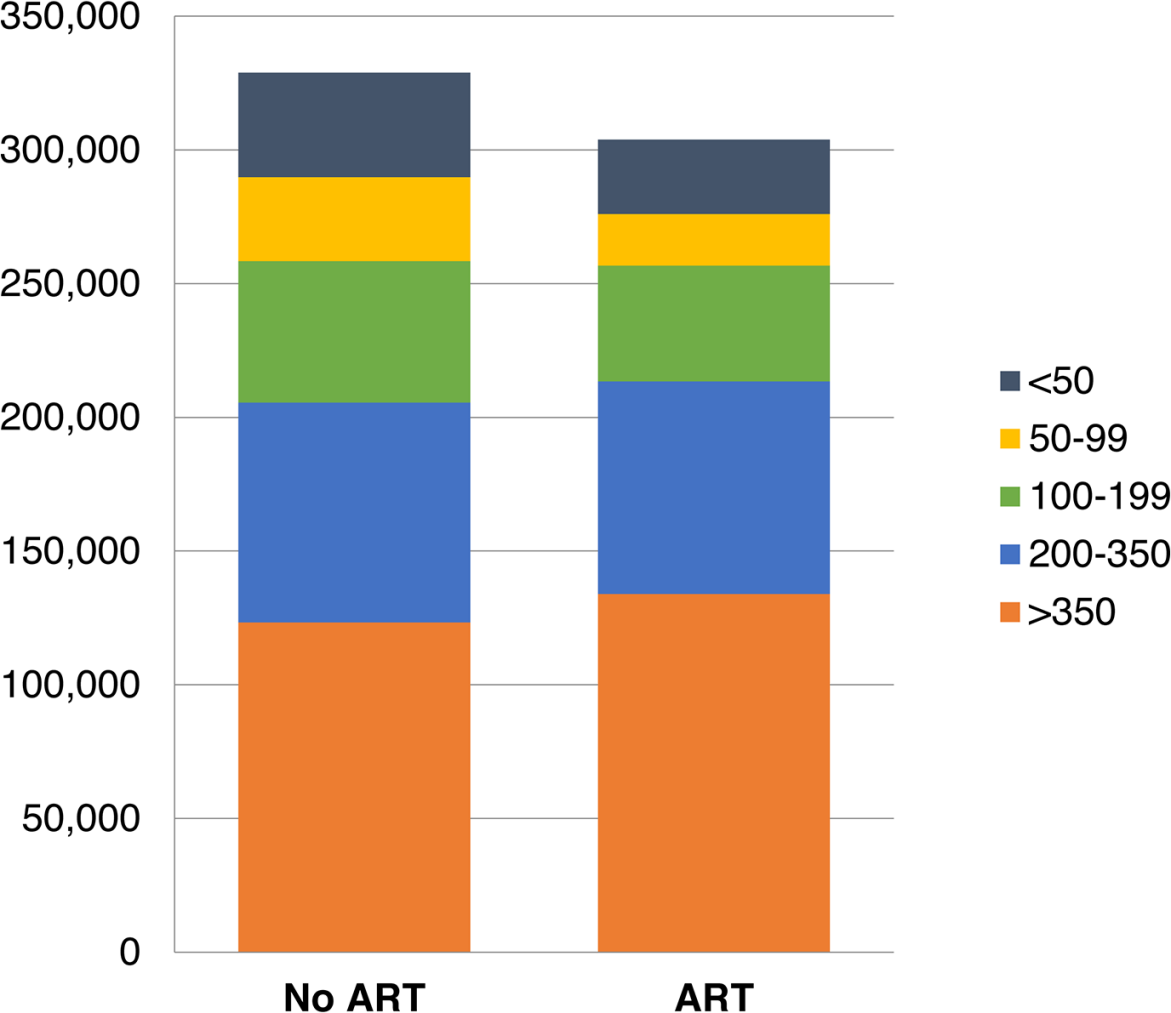
Total number of days absent due to HIV per CD4-cell-count-defined health state, 2003–2022.

With workplace ART provision, other changes are experienced by the workforce between 2003 and 2022. The total number of absent days due to HIV are estimated to be reduced by 8% (90% CrI 6%–10%), from 330,172 (90% CrI 297,729–367,723) to 303,897 (277,147–335,776) days, with 33% (90% CrI 26%–40%) fewer absenteeism days amongst employees with CD4 cell counts below 100 (Fig 4). The number of employees leaving employment for HIV-related reasons is estimated to decrease by 5% (90% CrI 3%–7%) to 3,626 (90% CrI 3,403–3,815) over 20 y, and the number of recruits is estimated to decrease by 2% (1%–3%) to 17,201 (16,454–17,912). Recruitment does not decrease further because of the large expansion of the company over this period (from 5,247 to 9,252 employees) and the considerable separations in the HIV-uninfected workforce.

### Total and Average Cost with and without ART

Without workplace ART provision, the undiscounted total cost of HIV to the company (including all healthcare, absenteeism, and turnover costs) over 20 y is estimated at US$296 million (90% CrI US$274–US$320 million) (Table 7), with the mean annual cost estimated to increase from US$13 million (90% CrI US$12–US$15 million) in the first 10 y to US$15 million (US$14–US$16 million) over 20 y, mostly due to increasing HIV prevalence. This translates to a mean annual cost per HIV-positive employee of US$13,271 (90% CrI US$12,101–US$14,522) over 20 y. With ART, over 98% of model projections suggest that these costs decrease: the total and mean annual costs are estimated to decrease by 5% (90% CrI 2%–8%) over 20 y, and the mean annual cost per HIV-positive employee by 9% (5%–13%). These savings are estimated to accrue from the first year of the ART programme onwards and to increase as the average CD4 cell count of HIV-positive employees on ART rises. Similar changes are seen with the discounted cost (S1 Fig). Moreover, ART is estimated to be cost-saving at even the lowest coverage level, as each employee on ART saves absenteeism, healthcare, and turnover costs that are greater than the per employee cost of ART.

**Table 7:** Total cost of HIV to company with and without ART programme and cost savings due to ART—main results and sensitivity analysis.

Scenario	No ART		ART		Savings from ART	
	By 2012	By 2022	By 2012	By 2022	By 2012	By 2022
**Cost of HIV to company: median (90% CrI) from probabilistic sensitivity analysis**						
***Undiscounted***						
Total cost (millions 2010 USD)	131 (118–147)	296 (274–320)	124 (112–140)	278 (256–299)	5% (2%–8%)	6% (1%–11%)
Mean annual cost (millions 2010 USD)	13 (12–15)	15 (14–16)	12 (11–14)	14 (13–15)	5% (2%–8%)	6% (1%–11%)
Mean annual cost per HIV-positive employee (2010 USD)	14,208 (12,982–15,509)	13,271 (12,101–14,522)	12,893 (11,903–13,862)	11,488 (10,601–12,218)	9% (5%–13%)	14% (7%–19%)
***Discounted***						
Total cost (millions 2010 USD)	155 (140–178)	269 (247–293)	148 (133–170)	253 (233–275)	5% (2%–7%)	6% (2%–10%)
Mean annual cost (millions 2010 USD)	16 (14–18)	13 (12–15)	15 (13–17)	13 (12–14)	5% (2%–7%)	6% (2%–10%)
Mean annual cost per HIV-positive employee (2010 USD)	16,936 (15,383–18,624)	12,045 (10,948–13,242)	15,409 (14,137–16,780)	10,492 (9,614–11,287)	9% (5%–13%)	13% (8%–18%)
**Sensitivity analysis: percent relative change in total undiscounted cost**						
Absenteeism reduced by 50%	−9%	−11%	−0.4%	−1%	−5%	−4%
Same absenteeism on ART as not on ART^1^	3%	3%	3%	4%	5%	5%
Same ART transition probabilities as public sector^2^	10%	13%	7%	9%	8%	10%
Same ART cost as public sector^3^	3%	3%	−1%	−4%	8%	12%
Change in inpatient cost: −50%	−6%	−7%	−6%	−6%	5%	5%
Change in inpatient cost: +50%	11%	12%	10%	10%	6%	8%
Change in outpatient cost: −50%	0.4%	−0.1%	−0.2%	−1%	6%	7%
Change in outpatient cost: +50%	5%	6%	5%	5%	5%	6%
Change in benefits: two times annual salary paid	−17%	−15%	−17%	−14%	5%	5%
Change in benefits: one times annual salary paid	−36%	−34%	−35%	−31%	4%	3%
Change in benefits: no benefits paid out	−56%	−52%	−54%	−48%	1%	−2%
Change in HIV-dependent separation rates: −20%	0.1%	3%	−1%	2%	6%	8%
Change in HIV-dependent separation rates: +20%	5%	2%	5%	2%	5%	6%
Change in HIV incidence: −50%	−17%	−22%	−17%	−22%	5%	6%
Change in HIV incidence: +50%	21%	26%	20%	25%	6%	7%
Change HIV incidence to 0.0001 and lower prevalence in starting population and recruits	−94%	−95%	−94%	−95%	5%	4%
**Additional scenarios: percent relative change in total undiscounted cost, 2013–2022**						
Test and treat^4^	—	—	—	0.2%	—	9%
Family treatment^5^	—	—	—	9%	—	1%

^1^By CD4-cell-count-defined health state.

^2^Based on [58] (public sector transition probabilities for first-line ART and first-line treatment failure only).

^3^US$277, the average per patient annual cost of adult ART in the public sector for 2015/2016, with 7.5% of patients assumed on second-line ART (based on [59], updated using April 2015 government tender drug costs).

^4^100% coverage with HCT; 100% initiation on ART regardless of CD4 cell count and clinical status; 100% retention on ART; no impact on HIV incidence.

^5^For every employee known to be HIV-positive, treatment is offered to one additional HIV-positive dependant on average.

### Average Cost and Savings by Item

Without ART provision, the largest components of the mean undiscounted annual cost of HIV to the company over 20 y are estimated to be benefit payments (53% of mean annual cost) and medical care costs (24%), followed by absenteeism (15%), and training and recruitment (8%) (Table 8). The cost of medical care is dominated by inpatient care (78% of medical care costs). Once ART is introduced, we estimate that benefit payments and medical care costs remain the largest contributors to the annual HIV costs (46% and 21%, respectively), whereas the cost of the ART programme itself is estimated to be comparatively small, at just 7% of the total.

**Table 8:** Annual undiscounted cost and savings by cost item, 2003–2022.

Cost Item	Annual Cost (Millions 2010 USD)				Savings from ART		
	No ART		ART		Total (Compared to No ART) (Millions 2010 USD)	Relative (Compared to No ART)	Percent of Total Saving^1^
	Cost	Percent of Total	Cost	Percent of Total			
**Medical care**	3.6 (3.3–3.9)	24% (22%–27%)	3.0 (2.7–3.4)	21% (15%–26%)	0.57 (0.28 to 0.78)	15% (−7% to 34%)	27% (8% to 37%)
Inpatient care	2.8 (2.6–3.0)	19% (17%–20%)	2.2 (2.0–2.4)	15% (11%–18%)	0.55 (0.41 to 0.68)	19% (−1% to 38%)	27% (11% to 35%)
Outpatient care	0.8 (0.6–1.1)	6% (4%–7%)	0.8 (0.6–1.1)	6% (4%–8%)	0.03 (−0.26 to 0.19)	2% (−41% to 32%)	0% (−14% to 11%)
**Absenteeism**	2.2 (2.0–2.4)	15% (13%–16%)	1.9 (1.8–2.1)	13% (10%–16%)	0.25 (0.20 to 0.30)	11% (−11% to 32%)	12% (4% to 22%)
**Benefits**	7.8 (7.1–8.7)	53% (50%–56%)	6.8 (6.1–7.5)	46% (33%–54%)	1.06 (0.69 to 1.52)	13% (−2% to 39%)	52% (8% to 66%)
**Training and recruitment**	1.2 (1.0–1.3)	8% (7%–8%)	1.0 (0.9–1.1)	6% (5%–8%)	0.19 (0.13 to 0.25)	15% (0.1% to 41%)	9% (0.1% to 12%)
**ART programme cost**	—	—	1.1 (0.7–1.6)	7% (4%–11%)	−1.10 (−1.61 to −0.71)	—	—
**Total**	14.8 (13.7–16.0)		13.9 (12.8–15.0)		0.95 (0.22 to 1.62)	14% (5% to 24%)	

Values are median (90% CrI) from the probabilistic sensitivity analysis.

^1^The values presented here are the mean (rather than median) (90% CrI) from the probabilistic sensitivity analysis.

Overall, the average undiscounted annual savings from scaling up ART coverage over 20 y are estimated to be US$950,215 (90% CrI US$220,879–US$1,616,104). The largest contribution to these estimated savings (52% of total savings) is the 13% decrease in benefit payments, followed by the 15% decrease in medical care costs (27% of total savings) (Table 8). Although the cost of training and recruitment is estimated to fall by 15% with ART, this makes up only 9% of annual savings, whilst the cost of absenteeism, which falls by 11%, is estimated to contribute 12% of savings. Without ART, the total undiscounted annual cost of HIV to the company is estimated to make up 3.6% (90% CrI 3.3%–3.9%) of total company payroll between 2003 and 2022, whereas with ART, this falls to 3.4% (3.1%–3.7%).

### Sensitivity and Uncertainty Analysis and Additional Scenarios

The univariate sensitivity analysis showed that total costs over 20 y are very sensitive to reductions in benefits paid for death and disability (−33%/66%) and changes in HIV incidence (±50%), as well as to using public sector data for CD4 cell count transition probabilities, reductions in absenteeism (−50%), and changes in inpatient cost (±50%) (Table 7). However, total costs do not change much if absenteeism by CD4 cell count category are assumed to be the same with and without ART or if the HIV-dependent separation rates (±20%) or outpatient costs (±50%) are changed. Equally, there is little change when ART costs from recent analyses of public sector ART provision are used [59]. Importantly, the only assumptions under which ART provision stops being cost-saving are if absenteeism is reduced by 50% (over both 10 and 20 y) or if no benefits are paid out (over 20 y only); under all other assumptions tested, ART still saves between 3% and 12% of total costs over 20 y. Finally, reducing HIV incidence as well as HIV prevalence in the starting population and recruits to low levels results in a much reduced HIV prevalence (1%) by 2022, representative of a low prevalence setting; in this scenario, the cost of HIV to the company reduces by 95% both without and with ART, with ART still saving 4% of costs.

The overall findings of the probabilistic sensitivity analysis agreed with the findings of the univariate sensitivity analysis, despite the wide ranges assigned to many model parameters, with over 98% of all model fits predicting that ART provision was cost-saving (Table 7). The analysis also reinforced the relative contribution of individual cost items to total cost (Table 8). The analysis of co-variance revealed that 69% of the variability in the total savings achieved with ART in the probabilistic sensitivity analysis (after 20 y and undiscounted) were explained by uncertainty in the costs of ART (64%), as well as in the difference between the upwards CD4-cell-count-defined health-state transition probabilities on ART compared to with wellness care (21%) (see S2 and S3 Figs), and in the outpatient costs on ART (15%). Interestingly, although the cost of ART is always a relatively small component of the total cost of HIV (5%–11%), it can contribute significantly to offsetting the cost savings achieved with ART, with the cost of ART cancelling out 53% (90% CrI 32%–87%) of all potential savings. Importantly, the model projections suggest ART will always be cost-saving if it costs less than US$2,057 per patient-year. The large dependence of the estimated cost savings on the difference between the ART and wellness care health-state transition probabilities suggests that ART will not be cost-saving if it has little benefit for disease progression on top of what is already achieved with wellness care.

The cost of HIV in the test and treat sensitivity scenario over 10 y (2013–2022) increases only marginally, by 0.2%, because of increased savings in terms of inpatient care, absenteeism, and benefit payments, which almost offsets the cost of the additional treatment occurring (Table 7). In the family treatment scenario, total cost with ART provision between 2013 and 2022 increases by 9%, but ART provision is still marginally cost-saving.

## Discussion

Using a dynamic health-state transition model, we conducted a cost–benefit analysis of an established ART programme operating in a number of coal mines in South Africa. Our analysis provides both a retrospective analysis of the programme between 2003 and 2010 and a projection of future developments based on the results of this retrospective analysis. When considering the impact of HIV on a company’s healthcare costs—as well as worker absenteeism, sickness and death benefits, and staff turnover—the introduction of ART to all eligible employees is cost-saving from the first year of the programme onwards. With ART provision, the total costs of HIV to the company over 20 y is estimated to be reduced by 6% (90% CrI 2%–11%), and the cost per HIV-positive employee is estimated to be reduced by 14% (7%–19%). Moreover, in our probabilistic sensitivity analysis, 98% of the 998 model fits (selected from amongst 20,000 model runs) confirm this cost savings. The biggest savings are due to reductions in the benefit payments for death and ill-health retirement, followed by a decrease in the cost of employee healthcare use. This finding that ART is cost-saving is robust to the uncertainty around the model parameters as well as to other changes in numerous parameters or assumptions, including if absenteeism is the same for employees on and off ART, if there are large reductions in benefit payments, and if HIV prevalence in the workforce is decreased to below 1%. The only instance where ART does not save costs over 20 y is if absenteeism in HIV-positive employees is reduced by 50% or if no benefits are paid out—though the latter strategy still saves costs over 10 y. In addition, a strategy of offering HIV testing to all employees and immediate ART to all HIV-positive employees also results in savings to the cost of the HIV programme, suggesting test and treat be recommended as a powerful intervention for companies trying to preserve their employees’ productivity. Offering ART to one family member for each HIV-positive employee, a generous assumption, reduces savings but is still cost-saving compared to no workplace ART provision.

Previous work has shown a heterogeneous impact of HIV on absenteeism and replacement cost. In a study of nearly a thousand firms operating in Africa in 1997, the impact of HIV on staff turnover was minimal, probably because of the lower HIV prevalence at that time, with difficulties in replacing professional staff being the most significant problem companies were facing [60]. In another study, the total cost per HIV infection to South African companies was estimated at US$2,094 to US$15,000 for an unskilled worker (in 2001 prices) and US$8,736 to US$65,000 for a manager [2]. A study of a Natal sugar mill found that on average 28 d were lost in each of the 2 y preceding retirement on grounds of ill-health and estimated that the cost of each HIV infection was roughly three times the employee’s annual salary per year [61]. Similarly, a large part of the savings in our analysis were due to a policy of benefits being paid to the employee or their family in the case of disability or death, which might not apply to other workplaces and might limit the generalisability of the results across workplaces and countries.

While our analysis adds to the body of knowledge on the economic impact of HIV and ART—through the use of detailed modelling incorporating a wealth of data on costs of HIV and ART outcomes from the same setting—our study nonetheless has limitations. First, it was limited to the direct cost of HIV to companies. In a previous study, the life insurer Metropolitan predicted that the indirect costs of HIV to business (including costs due to a loss in morale, legal costs, management costs, and labour consultation costs) could add up to 15% of the wage and salary budget by 2010 [62]. The provision of ART could improve morale and retention of skilled employees [5] as well as help safeguard the company’s license to mine [63]. Including this added indirect benefit of ART would have increased our savings from workplace ART provision. Second, we used an average drug cost for first-line and second-line ART that slightly underestimated the cost of ART in the later years of the projection, when more employees needed second-line ART, and did not stratify ART cost by time on treatment. However, since few HIV-positive employees were on second-line treatment throughout the projection period and the cost of ART was a small proportion of total costs, this underestimation is unlikely to change our findings. Third, data for some of the model inputs, such as transitions between certain CD4-cell-count-defined health states, was limited, resulting in uncertainty around some estimates. The effect of this uncertainty was included in our model projections as well as tested in our sensitivity analysis, and our results were found to be robust to changes along plausible ranges for these parameters. However, the deterministic nature of the model prevented it from capturing the full inherent variability present in this workforce. Lastly, we did not examine the impact of HIV prevention policies on the miners or their families.

Further work could involve evaluating the effects of prevention and treatment interventions on HIV incidence, including in the areas around the mines and in miners’ families, and the cost of new policies such as providing pre-exposure prophylaxis or increasing the accommodation of miners’ families in the vicinity of the mines, in compliance with the mining charter [63]. Finally, given our finding of the importance of the cost of ART in influencing cost savings, further reductions in the private sector cost of antiretroviral drugs remain crucial.

### Conclusion

Providing HIV care, including ART, in a workforce with high HIV prevalence and high resulting absenteeism and turnover can be cost-saving for the employer, with savings being greater at higher ART coverage, and might provide respite to the strained resources of large-scale public sector programmes. Beyond making good business sense, a company-level HIV care programme including ART could go a long way towards improving the strained labour relations in the South African mining sector, especially when improved access to healthcare extends to the entire community [64]. It is crucial that strategies such as those under study here are replicated in other companies in similar settings.

## Supporting Information

S1 Fig Total annual cost with and without ART (discounted and undiscounted), 2003–2022 (2010 USD). (TIF)

S2 Fig Results of analysis of co-variance: yearly cost of ART. (TIF)

S3 Fig Results of analysis of co-variance: difference between wellness care and ART transition probabilities. (TIF)

S1 Text Details on parameter estimation, probabilistic sensitivity analysis, and model calculations. (PDF)

## Abbreviations

ART: antiretroviral therapy
CrI: credibility interval
HCT: HIV counselling and testing
USD: US dollars
VL: viral load
