# Supplementary figures and images for: The Impact of Company-Level ART Provision to a Mining Workforce in South Africa: A Cost–Benefit Analysis
Source: PLoS Med. 2015 Sep 1;12(9):e1001869. doi: 10.1371/journal.pmed.1001869 (PMC4556678; doi:10.1371/journal.pmed.1001869)

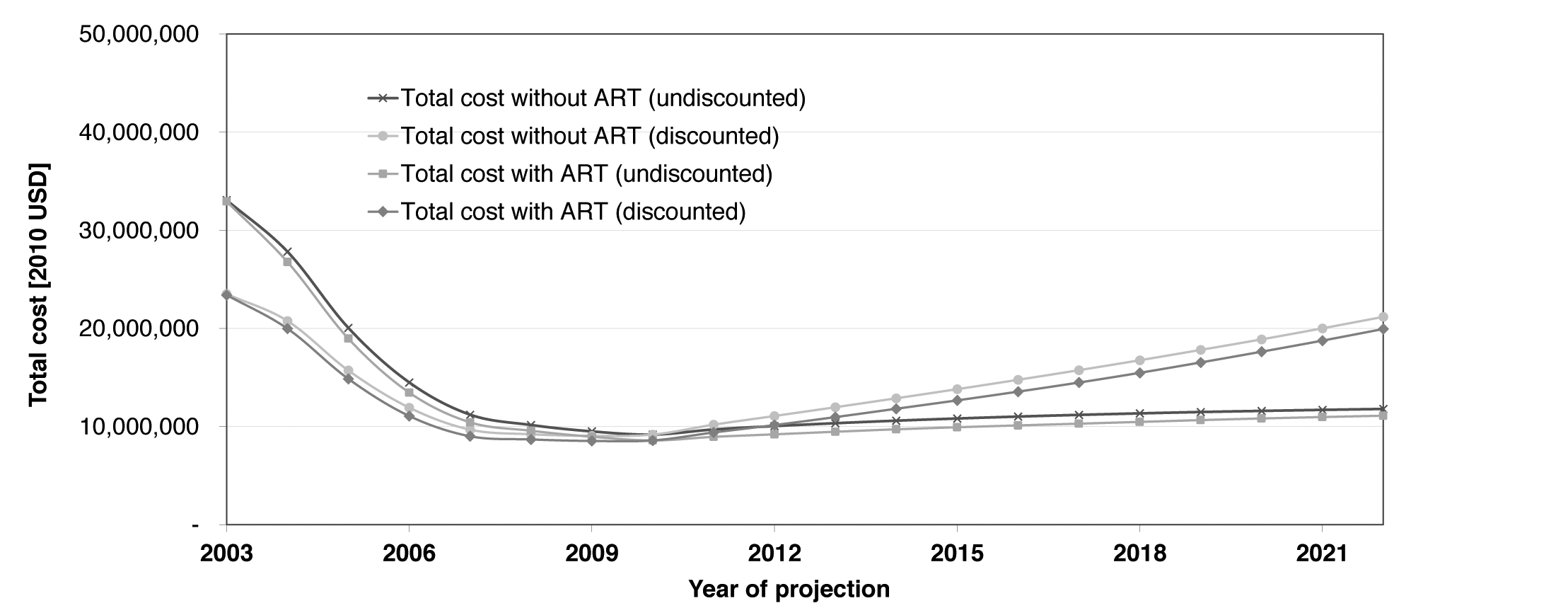

Supplement: S1 Fig — (TIF) [file pmed.1001869.s001.tif]

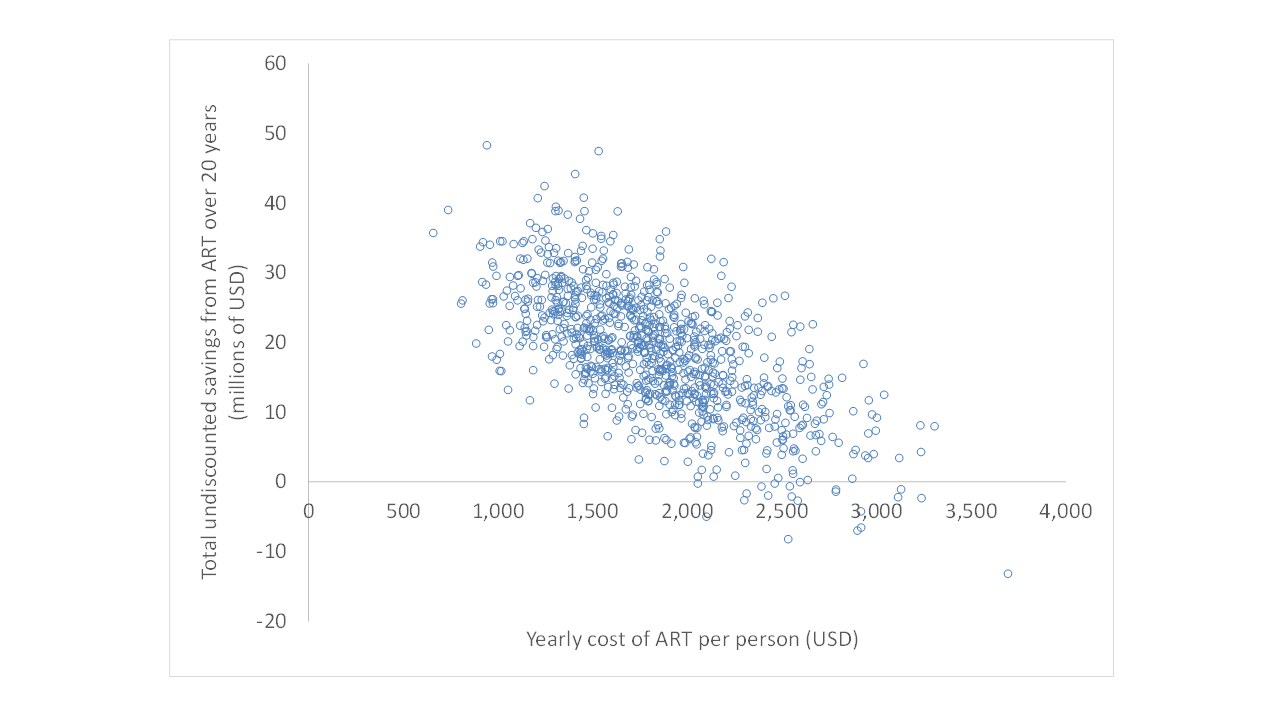

Supplement: S2 Fig — (TIF) [file pmed.1001869.s002.TIF]

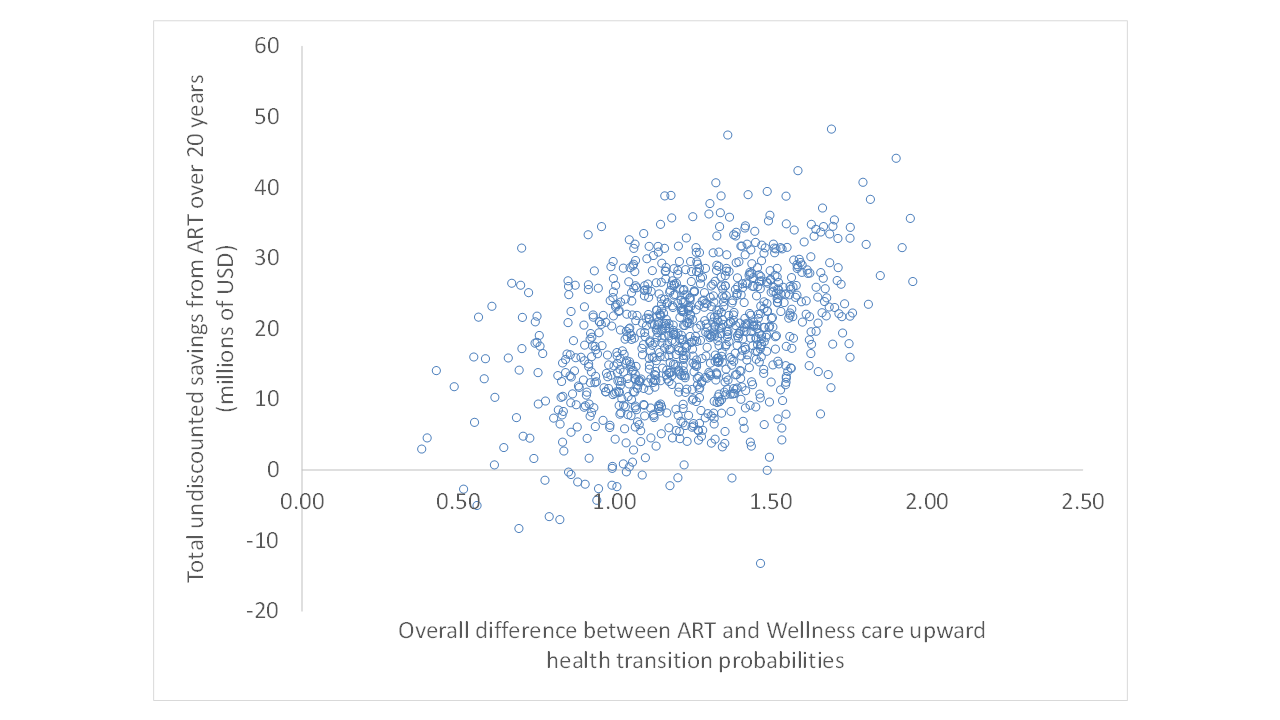

Supplement: S3 Fig — (TIF) [file pmed.1001869.s003.TIF]
