# Supplementary material for: The Impact of Company-Level ART Provision to a Mining Workforce in South Africa: A Cost–Benefit Analysis
Source: PLoS Med. 2015 Sep 1;12(9):e1001869. doi: 10.1371/journal.pmed.1001869 (PMC4556678; doi:10.1371/journal.pmed.1001869)
Supplement: S1 Text — (PDF) [file pmed.1001869.s004.pdf]

Company-level ART provision to employees is cost saving: A cost-benefit analysis of the impact of HIV and ART on a mining workforce in South Africa

Gesine Meyer-Rath, Jan Pienaar, Brian Brink, Andrew van Zyl, Debbie Muirhead, Alison Grant, Gavin Churchyard, Charlotte Watts, Peter Vickerman

Supplementary material

1. Additional details on parameter estimation and data sources

Table S1: Details of parameter estimation, level of stratification, and data sources

**Model input or assumption**	**Level of stratification**	**Source of data (2003-2010)**	**Method of estimation**	
			**2003-2010** (details of analysis given if parameter was analysed from company database; N = numerator, D = denominator)	**2011-2022**
**1. Changes in workforce**				
Workforce needed at end of year	Job grade, year	Company data	Data taken as is to calculate number of recruits or retrenchments required	Assumed to remain same as in 2010
Prevalence in recruits/ retrenchees[1]	Job grade, gender, year (for retrenchees, also by age)	Company data	N: All new employees with a positive first HIV result in the year of recruitment; D: all new employees with a positive or negative first HIV result in the year of recruitment	Assumed to remain same as in 2010
Distribution of recruits	Age group, gender, year	Company data (same as workforce distribution in database in 2003-2010)	N: Number of employees in database by year, job grade and gender/ age group; D: Total number of employees across all job grades, age groups and genders by year	Assumed same as average 2003-2010
Annual rate of promotion	Job grade, year	Company data for 2005/ 2006	All other years: assumed	
**2. Start population and coverage**				
Distribution of start population (all)	Age group, gender, job grade	Company data	Number of employees in database by 31 Dec 2002 by job grade, gender and age group	N/A (start year only)
HIV status of start population (all)	HIV status of those employees with an HIV test	Company data	Number of HIV-positive employees tested before 31 Dec 2002	

[1] If the workforce is set to be reduced during one year, the resulting number of recruits will be negative, signifying the number of people who will be retrenched, rather than recruited, during this year.

Model input or assumption	Level of stratification	Source of data (2003-2010)	Method of estimation	
			**2003-2010** (details of analysis given if parameter was analysed from company database; N = numerator, D = denominator)	**2011-2022**
Distribution of start population into CD4 cell count categories (HIV positives)	CD4 cell count category	No data	Same proportion assumed in each CD4 cell count strata	
Baseline HCT[2] coverage (full numbers)[3]	Age group, gender, job grade	Company data	Number of employees tested before 31 Dec 2002 by job grade, gender and age group	
**3. Costs**				
Average basic salary	Job grade	Company data (payroll)	Salaries in cost year (2006)	Real cost assumed constant over time
Incremental replacement cost for HIV-positive employees	Job grade	Interviews with company Human Resources department	Average cost per new employee by job grade in cost year (2006)	
Number of years that benefits get paid	None	Company benefit policy	Company policy	
Incremental inpatient/ outpatient cost for HIV-positive employees in cost year (2006)	Type of care (ART/ no ART), CD4 cell count category	Bottom-up cost analysis of company health services	Average cost per employee in cost year (2006); includes non-ARV drugs, non-ARV specific labs, patient contact time, other medical supplies, site programme cost, but no central management cost	
Annual per employee cost of ART[4] in cost year (2006)	CD4 cell count category	Bottom-up cost analysis of company health services	Average cost per employee in cost year (2006); includes central management cost for ART programme, ARV drug cost, ART-specific labs (CD4, VL5)	
Incremental absenteeism cost for HIV-positive employees	Type of care (ART/ no ART only), CD4 cell count category, job grade	Payroll data on sick leave days	Absent days/ shifts lost to sickness (sick leave) by health state in cost year (2006) multiplied by job-grade specific salary per day/ shift	
**4. Transitions between CD4 cell count categories**				
Transition probabilities	Type of care, CD4 cell count category	No care: public sector data (Ingle 2010[1]). All else: Company data	N: All employees with a CD4 cell count in one stratum in time period t who have a CD4 cell count in a different stratum in time period t+1; D: All employees with a CD4 cell count in one stratum in time period t	Assumed constant over time
**5. HIV incidence, coverage with testing, care and ART, treatment failure and retention**				

[2] HCT: HIV Counselling and Testing
[3] Coverage with all other care is set to zero at baseline
[4] ART: antiretroviral treatment
[5] VL: HIV viral load

Incidence	Job grade, CD4 cell count category[6], year	Change in HIV incidence over time fitted to company data on HIV incidence (Huber 2011[2]); job grade weights: company data; CD4 cell count category weights: assumed	HIV sero-conversion was assumed to occur at the mid-point between the first positive and the last previous negative HIV test. N: All employees with a calculated seroconversion date in one year; D: all employees with a negative HIV result and no seroconversion date in the previous year. This analysis excludes employees whose HIV test result was given as “unknown”.	Assumed same as average of 2008-2010
Coverage with HIV testing, wellness care and ART	Type of care, year and for ART, also CD4 cell count category	Company data	Model fitted to reported proportions of HIV-positive employees in each type of care	Assumed same as average of 2008-2010, except 1st line ART coverage from wellness which is used to achieve ~92% ART coverage of eligible population
Rate of treatment failure	Year (same for 1st and 2nd line)	Company data	N: Employees with a failure start date during cycle t; D: All employees on ART at the beginning of cycle t	Assumed same as average of 2008-2010
Loss to follow-up rate	Type of care, year	Company data	N: All employees with a care stop date (wellness care and ART only) during cycle t; D: All employees in wellness care and ART, respectively, at the beginning of cycle t	Assumed same as average of 2008-2010
**6. Separation rates**				
HIV-related	Type of separation, CD4 cell count category	Company data	Ill health, death, and other non-transfer separations were allocated to a CD4 cell count category using the last available CD4 cell count before exit from the workforce from the database.<br>N: All HIV-positive employees with an employment stop date by separation category and CD4 cell count category; D: All employee years in the same CD4 cell count category	Assumed constant over time
HIV-unrelated	Type of separation, job grade	Company data	N: All HIV-negative employees with an employment stop date by separation category and job grade; D: All employee years in the same job grade	Assumed constant over time

[6] Incidence is stratified by CD4 cell count category to allow the distribution of newly incident members of the Infected population into CD4 cell count categories. The values of the weights are 0.1, 0.2, 0.3, 0.5 and 1 for the categories >350, 200-350, 100-199, 50-99, <50 cells/microl, respectively.

Choice of parameters, distributions and shape parameters for probabilistic sensitivity analysis

Beta distributions were assigned to binomial events such as the proportion of individuals that experience either a HIV-related or non HIV-related separation, treatment failure or loss to ART retention in a specific time period, as well as the proportion of recruits that are newly HIV-infected in a specific year. Normal distributions were assigned to the number of absenteeism days experienced by individuals on or off ART for different CD4 cell count categories. Dirichlet distributions were assigned to the upward and downward transition probabilities between CD4 cell-count defined health states (further details below table). Lastly, because of the over dispersed nature of cost data, gamma distributions were assigned to the costs of in- and outpatient care for individuals on or off ART and to the cost of ART (which includes the cost of drugs, labs, other medical supplies, staff time, site programme cost and central management cost). Specific details on the parameter distributions used for each parameter and the justification for those assumptions are given in Table S2. Notation for the Gamma, normal and beta distribution is standard: Beta(α,β) gives a continuous approximation to a binomial distribution with α successes and β failures; N(μ,s^2) denotes a normal distribution with mean μ and standard deviation s; and G(a,b) denotes a gamma distribution with scale a and shape b.

Table S2: Summary of parameter values and the distributions chosen for their ranges

Parameter	Mean and 95% CI	Distribution	Reason for choice of distribution
**HIV incidence**			
2005	1.9% (1.5-2.3%)	Triangular with likeliest of 1.7% and min of 1.2% and maximum of 2.6%	HIV incidence varied across the years 2005 to 2009 with no obvious pattern, and so chose triangular distribution with most likely value being average across the years and the limits being the maximum and minimum of the 95% confidence intervals across the years.
2006	2.2% (1.8-2.6%)		
2007	1.6% (1.3-1.9%)		
2008	1.4% (1.2-1.7%)		
2009	1.6% (1.4-2.0%)		
**HIV-dependent separations per year**			
- Disability/Ill-health			
CD4>350			
CD4 200-350	0.76% (0.39-1.33%)	Beta(12,1563)	The distribution for all separation rates was estimated directly from the data used to derive it based on the number that separationed out of each CD4 health state (α in Beta(α,β)) from the total sample (α+β) for specific CD4 health state
CD4 100-200	1.51% (0.90-2.37%)	Beta(4,5122))	
CD4 50-100	2.09% (1.15-3.48%)	Beta(14,655)	
CD4<50	2.69% (0.99-5.76%)	Beta(6,217)	
- Death	13.8% (9.04-19.8%)	Beta(24,150)	
CD4>350			
CD4 200-350	1.90% (1.29-2.71%)	Beta(30,1546)	
CD4 100-200	3.94% (2.91-5.20%)	Beta(47,1146)	
CD4 50-100	9.25% (7.17-11.7%)	Beta(62,608)	
CD4<50	24.7% (19.2-30.9%)	Beta(55,167)	
- Other	67.25 (59.7-74.2%)	Beta(117,57)	
CD4>350			
CD4 200-350	6.98% (5.77-8.36%)	Beta(110,1466)	
CD4 100-200	8.13% (6.64-9.83%)	Beta(97,1095)	
CD4 50-100	8.51% (6.51-10.9%)	Beta(57,612)	
CD4<50	8.97% (5.56-13.5%)	Beta(20,203)	
	12.6% (8.10-18,5%)	Beta(22,152)	
**Non HIV-dependent separations per year**			
- Disability/Ill-health			
Job grade 1	0.66% (0.31-1.21%)	Beta(10,1501)	The distribution for all separation rates was estimated directly from the data used to derive it based on the number that separationed out of each job grade strata (α
Job grade 2	0.08% (0.02-0.20%)	Beta(4,5122)	
Job grade 3	0.21% (0.14-0.31%)	Beta(26,12,126)	
Job grade 4	0.24% (0.16-0.36%)	Beta(25,10,250)	

Job grade 5	0.09% (0.03-0.22%)	Beta(5,5405)	in Beta(α,β)) from the total sample (α+β) for specific job grade categories.
Job grade 6	0.03% (0.00-0.14%)	Beta(1,3864)	
- Death			
Job grade 1	0.99% (0.56-1.63%)	Beta(15,1496)	
Job grade 2	0.21% (0.11-0.38%)	Beta(11,5115)	
Job grade 3	0.57% (0.44-0.72%)	Beta(69,12083)	
Job grade 4	0.35% (0.25-0.48%)	Beta(36,10239)	
Job grade 5	0.28% (0.16-0.46%)	Beta(15,5395)	
Job grade 6	0.26% (0.12-0.48%)	Beta(10,3855)	
- Other			
Job grade 1	5.63%(4.52-6.91%)	Beta(85,1426)	
Job grade 2	1.50%(1.19-1.87%)	Beta(77,5049)	
Job grade 3	1.78% (1.55-2.03%)	Beta(216,11936)	
Job grade 4	8.53% (7.99-9.08%)	Beta(876,9399)	
Job grade 5	4.79% (4.23-5.39%)	Beta(259,5151)	
Job grade 6	5.92% (5.20-6.72%)	Beta(229,3636)	
- Retrenchment			
Job grade 1	0.26% (0.07-0.68%)	Beta(4,1507)	
Job grade 2	0.33% (0.19-0.53%)	Beta(17,5109)	
Job grade 3	0.09% (0.04-0.16%)	Beta(11,12141)	
Job grade 4	1.18% (0.98-1.41%)	Beta(121,10154)	
Job grade 5	0.57% (0.39-0.81%)	Beta(31,5379)	
Job grade 6	0.57% (0.36-0.86%)	Beta(22,3843)	
**Prevalence in recruits**			The distribution for yearly recruit prevalence estimates was estimated directly from data on the number of HIV positive recruits (32) out of the total number of new recruits tested (32+914) over years 2003 to 2010. The same prevalence was used for all years.
	3.50% (2.41-4.91%)	Beta(32,914)	
**Outpatient cost without ART (2010 ZAR)**			
CD4>350	1,353 (sd=1,656)	G(54,24)	As is standard practice, gamma distributions were assumed for all cost parameters because of their usual over dispersion. The shape (a) and scale (b) parameters for each distribution G(a,b) were derived using the method of moments where $a=(mean/SE)^2$ and $b=SE^2/mean$, with mean being the mean cost from the sample and SE being the standard error around the mean of the cost estimates in the sample. This was done for the cost data collected for in-patient as well as out-patient costs with each being stratified by the CD4 cell count of the individual. This was also done for the cost of ART for each individual. This was not stratified by CD4 count because the cost of ART did not vary by this variable within our data.
CD4 200-350	1,211 (sd=1675)	G(141,9)	
CD4 100-200	1,253 (sd=2391)	G(67,19)	
CD4 50-100	1,033 (sd=1331)	G(54,19)	
CD4<50	1,998 (sd=5647	G(11,181)	
**Inpatient cost without ART per year (2010 ZAR)**			
CD4>350	2,680 (sd=1,1827)	G(4.4,607)	
CD4 200-350	3,403 (sd=1,4206)	G(15,220)	
CD4 100-200	4,456 (sd=1,4432)	G(23,193)	
CD4 50-100	1,4658 (sd=32,709)	G(18,820)	
CD4<50	9,227 (sd=18,575)	G(22,425)	
**Outpatient cost with ART per year (2010 ZAR)**			
CD4>350	977 (sd=1,048)	G(704,1.4)	
CD4 200-350	993 (sd=1,255)	G(1015,1.0)	
CD4 100-200	963 (sd=1,280)	G(62,16)	
CD4 50-100	990 (sd=1,030)	G(373,2.7)	
CD4<50	1,176 (sd=1,905)	G(84,14)	
**Inpatient cost with ART per year (2010 ZAR)**			
CD4>350	1,779 (sd=6,415)	G(62,29)	
CD4 200-350	1,064 (sd=9,910)	G(19,57)	
CD4 100-200	1,755 (sd=1,1035)	G(2.8,637)	
CD4 50-100	2,426 (sd=1,1388)	G(18,132)	
CD4<50	9,327 (sd=27,070)	G(26,357)	
**Cost of ART per year (2010 ZAR)**			
	14,611	G(1034,14)	
**Absenteeism days per quarter without ART**			
CD4>350	4.59 (2.70-6.48)	N(4.6,0.56)	The distribution for number of absenteeism days was estimated directly from the data used to derive it based on the distribution of the number of absenteeism days reported by
CD4 200-350	3.78 (2.93-4.63)	N(3.8,0.25)	
CD4 100-200	6.06 (4.82-7.30)	N(6.1,0.37)	
CD4 50-100	9.63 (7.06-12.20)	N(9.6,0.76)	

CD4<50	13.80 (10.54-17.06)	N(13.8,0.96)	individuals with different CD4 cell counts. However, we did not sample from the full distribution across all individuals, but from the likely distribution around the mean for each CD4 cell count category. For this reason the standard error was used as the second parameter of the normal distribution and the mean was assumed to be normally distributed.
**Absenteeism days per quarter with ART**			
CD4>350	2.64 (2.20-3.08)	N(2.6,0.13)	
CD4 200-350	3.18 (2.83-3.53)	N(3.2,0.10)	
CD4 100-200	4.02 (3.48-4.56)	N(4.0,0.16)	
CD4 50-100	5.64 (4.45-6.83)	N(5.6,0.35)	
CD4<50	13.83 (10.79-16.87)	N(13.8,0.90)	
**Treatment failure**			
2003 to 2006	11.2% (8.47-14.3%)	Beta(53,422)	The distribution for treatment failure rate was estimated directly from the data used to derive it based on the proportion of individuals on treatment that failed treatment in each year. Rates were averaged for 2003 to 2006 and 2007 to 2010 to account for any variation over time. Rates after 2010 were assumed to be the same as for 2010.
2007 to 2010	8.09% (6.48-9.96%)	Beta(81,918)	
**Loss to retention from Wellness**			
2003 to 2006	0.67% (0.27-1.37%)	Beta(7,1045)	The distribution for each loss to retention rate was estimated directly from the data used to derive it based on the proportion of individuals on wellness care that were lost to follow up in each year. Rates were averaged for 2003 to 2006 and 2007 to 2010 to account for any variation over time. Rates after 2010 were assumed to be the same as for 2010.
2007 to 2010	1.74% (1.18-2.46%)	Beta(31,1754)	
**Loss to retention from ART or treatment failure**			
2003 to 2006	8.42% (6.08-11.29%)	Beta(40,435)	The distribution for each loss to retention was estimated directly from the data used to derive it based on the proportion of individuals on ART that were lost to follow up in each year. Rates were averaged for 2003 to 2006 and 2007 to 2010 to account for any variation over time. Rates after 2010 were assumed to be the same as for 2010.
2007 to 2010	9.89% (8.11-11.91%)	Beta(99.1001)	

For the CD4 transition probabilities, Dirichlet distributions were assumed for the alternative transitions from each baseline CD4 cell count strata over the next quarter- this is standard practice for variables that have multiple transition options. The data used to estimate the transition probabilities between health states is shown below from which the Dirichlet distributions were derived for the alternatives in each column. In this table, N denotes the number of 3-month periods of follow up and the numbers above each are the transitions that occurred over those time periods. Note that 1 = health state 1 (CD4 > 350 cells/microl), 2 = health state 2 (CD4 200-350 cells/microl), 3 = health state 3 (CD4 = 100-199 cells/microl), 4 = health state 4 (CD4 50-99 cells/microl), 5 = health state 5 (CD4 <50 cells/microl).

No care	from 1	2	3	4	5
to 1	3,852	-	-	-	-
2	193	2,516	-	-	-
3	33	159	1,914	-	-
4	5	24	81	706	-
5	10	26	43	74	915
**Total (N)**	**4,093**	**2,725**	**2,038**	**780**	**915**
**Wellness**	from 1	2	3	4	5
to 1	903	80	1	0	0
2	135	355	32	2	2
3	11	62	82	8	2
4	1	1	19	22	4
5	1	0	5	8	20
**Total (N)**	**1,051**	**498**	**139**	**40**	**28**
**ART**	from 1	2	3	4	5
to 1	1490	169	5	0	1
2	104	593	81	1	0
3	3	37	202	13	2
4	0	0	5	15	1
5	2	0	1	3	2
**Total (N)**	**1,599**	**799**	**294**	**32**	**6**

2. Additional details on methods

Inflation adjustment

Cost are given in 2010 constant USD. Even though inflating to the most recent year for which a Consumer Price Index (CPI) value is available is standard methodology, in the case of the healthcare cost in South Africa, the use of the general CPI for adjusting for inflation the expected value of a past cost analysis has its limit. Healthcare costs do not follow the general CPI, since salaries are subject to separate negotiations and drug prices (especially for antiretrovirals) have undergone dramatic downward developments since 2010. We think that inflating costs over a total of eight years (from 2006 to 2014) would have exaggerated them and render the final cost figures close to useless.

Model calculations

Standard methodology (Drummond 2005) suggests that the choice of health states in a health-state transition model be reflective of important differences in disease progression, or healthcare utilization and cost, or both, in order to best represent survival and cost associated with the disease or an intervention against it. In analysing the workplace data to decide on the number and definition of health states, we found differences in separations (morbidity and mortality) and promotion rates between job grades and age groups in all employees; and differences in incidence and separations between job grades, genders and age groups in HIV positive employees, as well as in absenteeism and cost between HIV-positive

employees with different CD4 cell count levels. This necessitated the number of categories that we used in the model, and which are given in more detail below.

Nomenclature

Model cycle	t
Calendar year	y
CD4-count defined health state	s for $s = 1…5$
Job grade	j for $j = 1…6$
Age group	a for $a = 1…3$
Gender	g for $g = 1,2$
Recruits/ retrenchees[7]	R
- HIV-positive recruits/ retrenchees	R^+
- HIV-negative recruits/ retrenchees	R^-
Total required workforce in year y	N_y
Total workforce in time step t	N_t
Separation rate per individual	d_m for $m = 1…6$
Total number of separations	D_m for $m = 1…6$
- non-HIV related separations	
- Death/ ill-health retirement (non-HIV related)	D_1
- Non-HIV disability	D_2
- Other (dismissed in absentia, etc; non-HIV related)	D_3
- HIV-related separations	
- Death/ ill-health retirement (HIV-related)	D_4
- HIV disability	D_5
- Other (dismissed in absentia, etc; HIV-related)	D_6
Retirees	E
HIV prevalence in recruits	P_R
Susceptibles -total	S
HIV incidence in year y	ir
Promotion rate from job grade j into job grade j+1 per year	pr
Aging rate from age group a into age group a+1 per year	ar
Infecteds -Total	I
- untested	I_u
- without care	I_n
- in Wellness care	I_w
- on first-line ART	I_{a1}
- in first-line failure	I_{ax1}
- on second-line ART	I_{a2}
- in second -line failure	I_{ax2}
- on any type of ART (I_{a1}, I_{a2}, I_{ax1}, or I_{ax2})	I_a
- not on ART (I_u, I_n, or I_{aw})	I_{na}
Yearly incremental coverage with	
- testing	c_t
- Wellness care from no care	$c_{n,w}$
- ART from no care	$c_{n,a1}$

[7] If the workforce is set to be reduced during one year, the resulting number of recruits will be negative, signifying the number of people who will be retrenched, rather than recruited, during this year.

- ART from Wellness	$c_{w,a1}$
- first-line ART from first-line treatment failure	$c_{ax1,a1}$[8]
- second-line ART from first-line treatment failure	$c_{ax1,a2}$
Transitions from CD4 cell count-defined health state s into health state s-x or s+x, where x = 1…4[9]	
Transition probability	tp
Treatment failure	tf
- first-line failure	tf_{a1}
- second-line failure	tf_{a2}
Loss to follow-up from	ltfu
- Wellness care	$ltfu_{w}$
- first-line treatment	$ltfu_{a1}$
- second-line treatment	$ltfu_{a2}$
- first-line failure	$ltfu_{ax1}$
- second-line failure	$ltfu_{ax2}$
Total inpatient cost	IC
- of patients on any type of ART (I_{a1}, I_{a2}, I_{ax1}, or I_{ax2})	IC_{a}
- of patients not on ART (I_{u}, I_{n}, or I_{aw})	IC_{na}
Total outpatient cost	OC
- of patients on any type of ART (I_{a1}, I_{a2}, I_{ax1}, or I_{ax2})	OC_{a}
- of patients not on ART (I_{u}, I_{n}, or I_{aw})	OC_{na}
Total absenteeism cost	AC
Mean days of absenteeism	AD
- of patients on any type of ART (I_{a1}, I_{a2}, I_{ax1}, or I_{ax2})	AD_{a}
- of patients not on ART (I_{u}, I_{n}, or I_{aw})	AD_{na}
Salary	
- daily salary	Sd
- annual salary	Sa
Incremental death benefits of HIV+ over HIV- employees	MB
Incremental disability benefits of HIV+ over HIV- employees	DB
Number of annual salaries used in the calculation of benefits	
- in case of death of an employee	MY
- in case of disability of an employee	DY
Total training and recruitment cost	TRC
Annual per employee training cost	C_{T}
Annual per employee recruitment cost	C_{R}
Total ART cost	TxC
Annual per employee ART cost	C_{ART}

Equations

1. Recruits/ retrenchees[10] (R)

[8] A small proportion (default value: 0.1%) of Infecteds in first-line treatment failure are assumed to move back to successful first-line treatment as a result of their viral load being re-suppressed after intensified adherence counselling and an improvement in adherence

[9] Despite s being included as a subscript to all transition probabilities, the transition probabilities are specific to the type of care

[10] If the workforce is set to be reduced during one year, the resulting number of recruits will be negative, signifying the number of people who will be retrenched, rather than recruited, during this year.

a) All recruits/ retrenchees

Recruits/ retrenchees in cycle t+1(in specific job grade, age and gender group) = (Workforce required in year y + all separations in cycle t + all retirements in cycle t - current workforce in cycle t) * proportion of required workforce in year y that needed in job grade * proportion of workforce in cycle t that is of this gender, age, and job grade out of all workforce in this job grade in cycle t

$$R_{t+1}(a,g,j)^{11} = (N_y + \sum D_{1...6t} + E_t - N_t) * N_y(j) / N_y * N_t(a,g,j) / N_t(j)$$

(Equation 1.1)

b) HIV-positive recruits/ retrenchees (R^+)

HIV-positive recruits/ retrenchees in cycle t+1 = Recruits/ retrenchees in cycle t+1 * prevalence in recruits[12] in year y (all in cycle t; all for the relevant age-, job grade- and gender-specific cohort)

$$R^+_{t+1}(s,a,g,j) = R_{t+1}(s,a,g,j) * P_R(a,g,j^{vi},y)$$

(Equation 1.2)

c) HIV-negative recruits/ retrenchees (R^-)

HIV-negative recruits/ retrenchees in cycle t+1 = Recruits/ retrenchees in cycle t+1 - HIV-positive recruits/ retrenchees in cycle t+1 (all in cycle t; all for the relevant age-, job grade- and gender-specific cohort)

$$R^-_{t+1}(a,g,j) = R_{t+1}(a,g,j) - R^+_{t+1}(a,g,j)$$

(Equation 1.3)

2. Susceptibles (S)

Susceptibles in cycle t+1 = Susceptibles in cycle t - HIV incident cases + HIV-ve recruits - non-HIV related separations - promotions to higher job grade - losses to older age group + gains from younger age group + promotions from lower job grade (all in cycle t; all for the relevant age-, job grade- and gender-specific cohort)

$$S_{t+1}(a,g,j) = S_t(a,g,j) - S_t(a,g,j) * ir(s,j,y)/4 + R^-_{t+1}(a,g,j) - S_t(a,g,j) * \sum d_{1,2,3}(j) - S_t(a,g,j) * (pr(j,y) + ar(a))/4 + S_t(a-1,g,j) * ar(a-1)/4 + S_t(a,g,j-1) * pr(j-1,y)/4$$

(Equation 2)

3a. Untested infected (I_u)

[11] For each parameter, variables in brackets denote the categories that the parameter was stratified by. Parameters without variables in brackets denote the total population or rate across all categories.

[12] If the number of recruits is positive in a year, prevalence in recruits is based on our analysis of workforce prevalence data (by year, gender and job grade); if it is negative (ie, retrenchees are being calculated), prevalence in recruits is based on general workforce prevalence in the model in the same year (by year, *age*, gender and job grade)

Untested infected in cycle t+1 = Untested infected in cycle t + (HIV+ve recruits + incident cases in cycle t) * (1 - testing coverage) - untested infected in cycle t * testing coverage - HIV related and unrelated separations - promotions to higher job grade - losses to older age group + gains from younger age group + promotions from lower job grade - transitions to lower health states[13] + transitions from higher health states (all in cycle t; all for the relevant health state-, age-, job grade- and gender-specific cohort)

$$\begin{aligned} I_{ut+1}(s,a,g,j) = \; & I_{ut}(s,a,g,j) + (R^{+}_{t}(s,a,g,j) + S_t(a,g,j) * ir(s,j,y)/4) * (1-c_t(y)/4) - I_{ut}(s,a,g,j) * c_t(y)/4 \\ & - I_{ut}(s,a,g,j) * \sum d_{1\ldots6}(s,j) \\ & - I_{ut}(s,a,g,j) * (pr(j,y) + ar(a))/4 + I_{ut}(s,a-1,g,j) * ar(a-1)/4 + I_{ut}(s,a,g,j-1) * pr(j-1,y)/4 \\ & - I_{ut}(s,a,g,j) * \sum_{all\ x\geq1} tp_{u,s,s-x} + \sum_{all\ x\geq1} I_{ut}(s+x,a,g,j) * tp_{u,s+x,s} \end{aligned}$$

(Equation 3.1)

3b. Infected without care (I_n)

Infected without care in cycle t+1 = Infected without care in cycle t + (HIV+ve recruits + incident cases in cycle t) * testing coverage - coverage with Wellness and ART + infected in Wellness care, first-line ART, first-line treatment failure, second-line ART and second-line treatment failure who are lost to care - HIV related and unrelated separations - promotions to higher job grade - losses to older age group + gains from younger age group + promotions from lower job grade - transitions to lower health states[xiii] + transitions from higher health states (all in cycle t; all for the relevant health state-, age-, job grade- and gender-specific cohort)

$$\begin{aligned} I_{nt+1}(s,a,g,j) = \; & I_{nt}(s,a,g,j) + (R^{+}_{t}(s,a,g,j) + S_t(a,g,j) * ir(s,j,y)/4) * c_t(y)/4 - I_{nt}(s,a,g,j) * (c_{n,w}(y)/4 + \\ & c_{n,a1}(s,y)/4) \\ & + I_{wt}(s,a,g,j) * ltfu_w(y,g)/4 + I_{a1t}(s,a,g,j) * ltfu_{a1}(y)/4 + I_{ax1t}(s,a,g,j) * ltfu_{ax1}(y)/4 \\ & + I_{a2t}(s,a,g,j) * ltfu_{a2}(y)/4 + I_{ax2t}(s,a,g,j) * ltfu_{ax2}(y)/4 \\ & - I_{nt}(s,a,g,j) * \sum d_{1\ldots6}(s,j) \\ & - I_{nt}(s,a,g,j) * (pr(j,y) + ar(a))/4 + I_{nt}(s,a-1,g,j) * ar(a-1)/4 + I_{nt}(s,a,g,j-1) * pr(j-1,y)/4 \\ & - I_{nt}(s,a,g,j) * \sum_{all\ x\geq1} tp_{n,s,s-x} + \sum_{all\ x\geq1} I_{nt}(s+x,a,g,j) * tp_{n,s+x,s} \end{aligned}$$

(Equation 3.2)

3c. Infected and covered by Wellness care (I_w)

Infected in Wellness care in cycle t+1 = Infected in Wellness care in cycle t + infected without care in cycle t covered with Wellness care - coverage with 1st line ART - HIV related and unrelated separations - loss to follow-up - promotions to higher job grade - losses to older age group + gains from younger age

[13] In both the untested and the without care populations, transitions between CD4 cell count categories are unidirectional, with the only possible movement (for those who do not stay in the same health state) being to lower health states (see Table 2 in main paper)

group + promotions from lower job grade - transitions to higher and lower health states + transitions from lower and higher health states (all in cycle t; all for the relevant health state-, age-, job grade- and gender-specific cohort)

$$\begin{aligned} \mathbf{I_{wt+1}(s,a,g,j)} = {} & \mathbf{I_{wt}(s,a,g,j) + I_{nt}(s,a,g,j) * c_{n,w}(y)/4 - I_{wt}(s,a,g,j) * c_{w,a1}(s,y)/4} \\ & \mathbf{- I_{wt}(s,a,g,j) * \Sigma d_{1...6}(s,j) - I_{wt}(s,a,g,j) * ltfu_{w}(y,g)/4} \\ & \mathbf{- I_{wt}(s,a,g,j) * (pr(j,y) + ar(a))/4 + I_{wt}(s,a\text{-}1,g,j) * ar(a\text{-}1)/4 + I_{wt}(s,a,g,j\text{-}1) * pr(j\text{-}1,y)/4} \\ & \mathbf{- I_{wt}(s,a,g,j) * \Sigma_{all\ x\geq 1}\ tp_{w,s,s+x} - I_{wt}(s,a,g,j) * \Sigma_{all\ x\geq 1}\ tp_{w,s,s-x}} \\ & \mathbf{+ \Sigma_{all\ x\geq 1} I_{wt}(s+x,a,g,j) * tp_{w,s+x,s} + \Sigma_{all\ x\geq 1}\ I_{wt}(s\text{-}x,a,g,j) * tp_{w,s-x,s}} \end{aligned}$$

(Equation 3.3)

3d. Infected and covered by 1st line ART (I_{a1})

Infected on 1st line ART in cycle t+1 = Infected on 1st line ART in cycle t + infected without care in cycle t covered with 1st line ART + infected in Wellness care in cycle t covered with 1st line ART - 1st line treatment failure - HIV related and unrelated separations - loss to follow-up - promotions to higher job grade - losses to older age group + gains from younger age group + promotions from lower job grade - transitions to higher and lower health states + transitions from lower and higher health states (all in cycle t; all for the relevant health state-, age-, job grade- and gender-specific cohort)

$$\begin{aligned} \mathbf{I_{a1t+1}(s,a,g,j)} = {} & \mathbf{I_{a1t}(s,a,g,j) + I_{nt}(s,a,g,j) * c_{n,a1}(s,y)/4 + I_{wt}(s,a,g,j) * c_{w,a1}(s,y)/4 - I_{a1t}(s,a,g,j) * tf_{a1}(y)/4} \\ & \mathbf{- I_{a1t}(s,a,g,j) * \Sigma d_{1...6}(s,j) - I_{a1t}(s,a,g,j) * ltfu_{a1}(y)/4} \\ & \mathbf{- I_{a1t}(s,a,g,j) * (pr(j,y) + ar(a))/4 + I_{a1t}(s,a\text{-}1,g,j) * ar(a\text{-}1)/4 + I_{a1t}(s,a,g,j\text{-}1) * pr(j\text{-}1,y)/4} \\ & \mathbf{- I_{a1t}(s,a,g,j) * \Sigma_{all\ x\geq 1} tp_{a1,s,s+x} - I_{a1t}(s,a,g,j) * \Sigma_{all\ x\geq 1}\ tp_{a1,s,s-x}} \\ & \mathbf{+ \Sigma_{all\ x\geq 1}\ I_{a1t}(s+x,a,g,j) * tp_{a1,s+x,s} + \Sigma_{all\ x\geq 1}\ I_{a1t}(s\text{-}x,a,g,j) * tp_{a1,s-x,s}} \end{aligned}$$

(Equation 3.4)

3e. Infected in 1st line treatment failure (I_{ax1})

Infected in 1st line treatment failure in cycle t+1 = Infected in 1st line treatment failure in cycle t + infected on 1st line treatment in cycle t * 1st line treatment failure rate - coverage with 2nd line ART - re-coverage with 1st line ART - HIV related and unrelated separations - loss to follow-up - promotions to higher job grade - losses to older age group + gains from younger age group + promotions from lower job grade - transitions to higher and lower health states + transitions from lower and higher health states (all in cycle t; all for the relevant health state-, age-, job grade- and gender-specific cohort)

$$\begin{aligned} \mathbf{I_{ax1t+1}(s,a,g,j)} = {} & \mathbf{I_{ax1t}(s,a,g,j) + I_{a1t}(s,a,g,j) * tf_{a1}(y)/4 - I_{ax1t}(s,a,g,j) * (c_{ax1,a2}(y)/4 + c_{ax1,a1}(y)/4)} \\ & \mathbf{- I_{ax1t}(s,a,g,j) * \Sigma d_{1...6}(s,j) - I_{ax1t}(s,a,g,j) * ltfu_{ax1}(y)/4} \\ & \mathbf{- I_{ax1t}(s,a,g,j) * (pr(j,y) + ar(a))/4 + I_{ax1t}(s,a\text{-}1,g,j) * ar(a\text{-}1)/4 + I_{ax1t}(a,g,j\text{-}1) * pr(j\text{-}1,y)/4} \end{aligned}$$

$$- I_{ax1t}(s,a,g,j) * \sum_{all\ x\geq 1} tp_{ax1,s,s+x} - I_{ax1t}(s,a,g,j) * \sum_{all\ x\geq 1} tp_{ax1,s,s-x}$$
$$+ \sum_{all\ x\geq 1} I_{ax1t}(s+x,a,g,j) * tp_{ax1,s+x,s} + \sum_{all\ x\geq 1} I_{ax1t}(s-x,a,g,j) * tp_{ax1,s-x,s}$$

(Equation 3.5)

3f. Infected and covered by 2nd line ART (I_{a2})

Infected on second line ART in cycle t+1 = Infected on 2nd line ART in cycle t + 1st line treatment failure covered with 2nd line treatment - 2nd line treatment failure - HIV related and unrelated separations - loss to follow-up - promotions to higher job grade - losses to older age group + gains from younger age group + promotions from lower job grade - transitions to higher and lower health states + transitions from lower and higher health states (all in cycle t; all for the relevant health state-, age-, job grade- and gender-specific cohort)

$$I_{a2t+1}(s,a,g,j) = I_{a2t}(s,a,g,j) + I_{ax1t}(s,a,g,j) * c_{ax1,a2}(y)/4 - I_{a2t}(s,a,g,j) * tf_{a2}(y)/4$$
$$- I_{a2t}(s,a,g,j) * \sum d_{1...6}(s,j) - I_{a2t}(s,a,g,j) * ltfu_{a2}(y)/4$$
$$- I_{a2t}(s,a,g,j) * (pr(j,y) + ar(a))/4 + I_{a2t}(s,a-1,g,j) * ar(a-1)/4 + I_{a2t}(s,a,g,j-1) * pr(j-1,y)/4$$
$$- I_{a2t}(s,a,g,j) * \sum_{all\ x\geq 1} tp_{a2,s,s+x} - I_{a2t}(s,a,g,j) * \sum_{all\ x\geq 1} tp_{a2,s,s-x}$$
$$+\sum_{all\ x\geq 1} I_{a2t}(s+x,a,g,j) * tp_{a2,s+x,s} + \sum_{all\ x\geq 1} I_{a2t}(s-x,a,g,j) * tp_{a2,s-x,s}$$

(Equation 3.6)

3g. Infected in 2nd line treatment failure (I_{ax2})

Infected in 2nd line treatment failure in cycle t+1 = Infected in 2nd line treatment failure in cycle t + infected on 2nd line treatment in cycle t * 2nd line treatment failure rate - HIV related and unrelated separations - loss to follow-up - promotions to higher job grade - losses to older age group + gains from younger age group + promotions from lower job grade - transitions to higher and lower health states + transitions from lower and higher health states (all in cycle t; all for the relevant health state-, age-, job grade- and gender-specific cohort)

$$I_{ax2t+1}(s,a,g,j) = I_{ax2t}(s,a,g,j) + I_{a2t}(s,a,g,j) * tf_{a2}(y)/4$$
$$- I_{ax2t}(s,a,g,j) * \sum d_{1...6}(s,j) - I_{ax2t}(s,a,g,j) * ltfu_{ax2}(y)/4$$
$$- I_{ax2t}(s,a,g,j) * (pr(j,y) + ar(a))/4 + I_{ax2t}(s,a-1,g,j) * ar(a-1)/4 + I_{ax2t}(s,a,g,j-1) * pr(j-1,y)/4$$
$$- I_{ax2t}(s,a,g,j) * \sum_{all\ x\geq 1} tp_{ax2,s,s+x} - I_{ax2t}(s,a,g,j) * \sum_{all\ x\geq 1} tp_{ax2,s,s-x}$$
$$+ \sum_{all\ x\geq 1} I_{ax2t}(s+x,a,g,j) * tp_{ax2,s+x,s} + \sum_{all\ x\geq 1} I_{ax2t}(s-x,a,g,j) * tp_{ax2,s-x,s}$$

(Equation 3.7)

4. Total separations (D)

a) For HIV-negative employees

$$\sum_{all\ j} D_{m1,2,3t}(j) = S_t(j) * \sum d_{1,2,3}(j)$$

(Equation 4.1)

b) For HIV-positive employees

$$\sum_{all\ j\ and\ s} D_{m1...6t}(s) = I_t(s) * \sum d_{1...6}(s,j)$$

(Equation 4.2)

5. Cost

a) Inpatient cost (IC)

$$IC = \sum_{all\ s} I_a(s) * IC_a(s) + \sum I_{na}(s) * IC_{na}(s)$$

(Equation 5.1)

b) Outpatient cost (OC)

$$OC = \sum_{all\ s} I_a(s) * OC_a(s) + \sum I_{na}(s) * OC_{na}(s)$$

(Equation 5.2)

c) Absenteeism cost (AC)

$$AC = \sum_{all\ j\ and\ s} I_a(j,s) * AD_a(s) * Sd(j) + \sum I_{na}(j,s) * AD_{na}(s) * Sd(j)$$

(Equation 5.3)

d) Incremental replacement cost due to HIV

i. Death benefits (MB)

$$MB = \sum_{all\ j\ and\ s} I(j,s) * (d_4(s) - d_1(j))^{14} * Sa(j) * MY$$

(Equation 5.4)

ii. Disability benefits (DB)

$$DB = \sum_{all\ j\ and\ s} I(j,s) * (d_5(s) - d_2(j)) * Sa(j) * DY$$

(Equation 5.5)

iii. Training and recruitment cost (TRC) (only in cycles in which R_t is >0)

$$TRC = \sum_{all\ j\ and\ s} I(j,s) * (d_{4,5,6}(s) - d_{1,2,3}(j)) * (C_T(j) + C_R(j))$$

(Equation 5.6)

e) ART cost (TxC)

$$TxC = \sum_{all\ s} I_a(s) * C_{ART}$$

(Equation 5.7)

References

1. Drummond 2005: Drummond MF, Sculpher MJ, Torrance GW, O'Brien BJ, Stoddart GL (2005) Methods for the Economic Evaluation of Health Care Programmes. New York: Oxford University Press, 3 ed.

[14] In order to calculate the incremental separations of HIV-positive employees over HIV-negative employees, we subtract the separation rates in HIV-negative employees from those in HIV-positive employees

2. Ingle SM (2010) Modelling waiting times for HIV antiretroviral therapy in South Africa: Patient management, outcomes and treatment rationing strategies in the Free State Province. PhD thesis, University of Bristol.

3. Huber A, Pienaar J, Innes C, Felix M, Churchyard GJ, Hoffmann CJ, Fielding KL, Latka MH (2011) Impact of universal VCT on population HIV incidence within a cohort of South African coal miners. International AIDS Society Conference 2011
